# Young Adults’ Responses to an African and US-Based COVID-19 Edutainment Miniseries: Real-Time Qualitative Analysis of Online Social Media Engagement

**DOI:** 10.2196/30449

**Published:** 2021-10-29

**Authors:** Venetia Baker, Georgia Arnold, Sara Piot, Lesedi Thwala, Judith Glynn, James Hargreaves, Isolde Birdthistle

**Affiliations:** 1 Department of Population Health Faculty of Epidemiology and Population Health The London School of Hygiene & Tropical Medicine London United Kingdom; 2 The MTV Staying Alive Foundation London United Kingdom; 3 Faculty of Public Health and Policy The London School of Hygiene & Tropical Medicine London United Kingdom

**Keywords:** COVID-19, adolescents, young people, social media, edutainment

## Abstract

**Background:**

In April 2020, as cases of the novel COVID-19 spread across the globe, MTV Staying Alive Foundation created the educational entertainment miniseries *MTV Shuga: Alone Together*. In 70 short episodes released daily on YouTube, *Alone Together* aimed to disseminate timely and accurate information to increase young people’s knowledge, motivation, and actions to prevent COVID-19.

**Objective:**

We sought to identify *Alone Together* viewer’s perspectives on the global COVID-19 pandemic and national lockdowns by examining the words, conversations, experiences, and emotions expressed on social media in response to the *Alone Together* episodes. We also assessed how viewers used the series and its online community as a source of support during the global pandemic.

**Methods:**

A total of 3982 comments and 70 live chat conversations were extracted from YouTube between April and October 2020 and analyzed through a data-led inductive thematic approach. Aggregated demographic and geographical data were collected using YouTube Analytics.

**Results:**

The miniseries had a global reach across 5 continents, with a total of 7.7 million views across MTV Shuga platforms. The series had over 1 million views over 70 episodes on YouTube and an average of 5683 unique viewers per episode on YouTube. The dominant audience was adults under the age of 35 years and women. Across diverse countries such as Nigeria, Ghana, the United States, and the UK, viewers believed that COVID-19 was serious and expressed that it was socially responsible to follow public health measures. Storylines of the series about the impact of self-isolation on mental health, exposure to violence in lockdowns, and restricted employment opportunities due to the pandemic resonated with young viewers. Tuning in to the miniseries provided viewers with reliable information, entertainment, and an online community during an isolating, confusing, and worrying time.

**Conclusions:**

During the first wave of COVID-19, viewers from at least 53 countries connected on social media via the MTV miniseries. The analysis showed how digitally connected people under the age of 35 years, predominantly women, felt compelled to follow COVID-19 safety measures despite the pandemic’s impact on their social, educational, and financial needs. Viewers used social media to reach out to fellow viewers for advice, solace, support, and resources. Organizations, governments, and individuals have been forced to innovate during the pandemic to ensure people can access services safely and remotely. This analysis showed that women under 35 years of age were especially receptive to receiving support from online communities and media services. Peer influence and support online can be a powerful public health tool as people have a great capacity to influence each other and shape norms around public health. However, online services are not accessible to everyone, and COVID-19 has increased disparities between digitally connected and unconnected younger adults.

## Introduction

The quick dissemination of accurate information has been an essential priority to educate the global public about the transmission and prevention of the novel SARS-CoV-2. Mass media is a cost-effective method for reaching and informing large populations quickly, like the TV and radio dramas used to educate captive audiences about the HIV epidemic [1-5]. During the COVID-19 pandemic, social media platforms used algorithms to direct its over 2 billion users toward trusted sources of information and flag misinformation, though it has also been responsible for fueling rumors, hoaxes, and misinformation about COVID-19 [6,7]. Younger adults (aged <35 years) rely particularly heavily on social media and online news platforms during the COVID-19 pandemic compared with older adults who additionally rely on close social partners and more traditional media [8].

In April 2020, as the novel COVID-19 spread across the globe, MTV Staying Alive Foundation (SAF) quickly produced an online “edutainment” miniseries that aimed to disseminate timely and accurate information to increase young people’s knowledge, motivation, and actions to reduce the spread of SARS-CoV-2. The series was called *MTV Shuga: Alone Together* and included storylines about the pandemic’s impact on employment, sexual and intimate partner violence, and mental health. MTV Shuga is MTV SAF’s flagship behavior change campaign, including a multiseason TV drama series, which integrates health messaging into storylines that reflect the lives and experiences of youth audiences. Social media engagement is a crucial element of the MTV Shuga campaign.

The *Alone Together* series featured characters from previous MTV Shuga series experiencing challenges with COVID-19 and lockdown measures across 6 countries: Cote d'Ivoire, Kenya, Nigeria, South Africa, Botswana, and the United States. MTV Shuga producers and writers formed a real-time collaboration with epidemiologists and social science researchers to create scripts and storylines that incorporated the most current COVID-19 science and country-specific guidance. Five-minute episodes were released daily, freely available on the internet using social media channels, where viewers could make comments as they watched.

Here we will present an analysis of the online viewer engagement with the *MTV Shuga: Alone Together* miniseries on YouTube. The analysis had 2 main objectives. First, by examining how viewers reacted to the miniseries storylines, we explored people’s perspectives on the burgeoning COVID-19 pandemic and national lockdown measures happening in real life. Second, we examined how viewers used an online community of virtual peers and fictional characters as a source of support during the pandemic.

## Methods

### Data Extraction

MTV SAF released 70 short episodes in daily instalments on YouTube, the MTV Shuga website, and Facebook Live. We extracted data from the YouTube comment section and the live chat stream within 2 weeks after episodes had aired during the first phase of data extraction. We then conducted the second data collection stage of extraction in October 2020, where 3982 comments and 70 live chats were extracted from YouTube as the series steadily gained more viewers and engagement. The series was broadcast in English; French subtitles were provided on a separate MTV Shuga YouTube channel. Comments that were not in English were excluded so no comments from the episodes with French subtitles were selected.

### Consent and Ethics

All comments and live chats extracted are public content, freely available on the internet, and we did not seek research ethics approval. We sought to treat the material responsibly, avoiding the publication of identifying information (ie, usernames) and deductive disclosure. We did not seek to identify the demographic data of individuals who posted in the comment section and live chat discussions; however, we collected aggregate demographic data from the series viewers who consented to YouTube’s terms and conditions.

### Analysis

We used NVivo software version 12 (QSR International) for data extraction, management, and analysis of the YouTube comments and live chats. As the data were unstructured, we chose to conduct data-led research using an inductive thematic approach [9]. In the first stage of data extraction and analysis, we analyzed the data in batches and compiled weekly reports to describe themes and codes emerging from the online comments. The reports were discussed weekly with the production and research team to identify viewer reactions and areas in which subsequent scripts and social media engagement could maximize the series’ impact on viewers. This timeline captured audience participation while allowing a rapid analysis that encapsulated the evolving global trajectory and epidemiological understanding of the COVID-19 pandemic, impacting country responses and public health messaging.

The comments from the first phase of analysis fell into 5 different categories (Textbox 1).

**Categories of comments.**

**Hooks**

Comments about the entertainment elements of the show that made viewers want to engage in *Alone Together*, including music, actors, country representation, drama, and suspense.

**COVID-19 Storylines**

Comments about COVID-19 and lockdown-related storylines, including stories about COVID-19, isolation, social distancing, domestic violence in lockdown, and economic hardships caused by COVID-19 restrictions.

**Non–COVID-19 Storylines**

Comments about storylines that were not COVID-19 or lockdown specific, including stories about romance, racial justice, and family conflicts.

**Viewers Experiences**

Comments that revealed viewers’ personal experience with the pandemic and their coping mechanism.

**Irrelevant Data**

Comments about self-promotion, spam, or random topic that did not relate to *Alone Together* or the COVID-19 pandemic.

Once the series was complete, we conducted a more detailed data analysis with a narrower scope. Using data extracted between April and October 2020, we selected data that fell into the categories of comments about COVID-19–related storylines and viewers’ personal experiences and coping mechanisms during the pandemic. Using a thematic analysis, we identified patterns in viewers’ comments that appeared across episodes. We familiarized ourselves with the data by reading through the comments and live chats from all episodes, then coded the data, and grouped codes into themes. Themes were finalized based on their reoccurrence throughout the data set and if they fell within the scope of the second analysis. We compiled a coding frame to demonstrate overarching patterns and categories across all episodes and then summarized and selected quotes to illustrate each theme.

We used YouTube Analytics to collect data about *Alone Together* viewers’ ages, gender, and locations in aggregate, and the number of views and minutes watched for each episode.

## Results

### Viewers’ Characteristics

As of April 2021, the *Alone Together* series had 1 million views over 70 episodes on YouTube, with an average of 5683 unique viewers per episode and over 6.7 million views on Facebook Live (Multimedia Appendix 1). On YouTube, the series had a large reach across 5 continents and was most popular in Nigeria, Ghana, the United States, the United Kingdom, Kenya, Canada, and South Africa. The majority of the audience on the English YouTube channel (Table 1) were aged 18-24 years (529,856/974,000, 54.40%, viewers) or 25-34 years old (394,470/974,000, 40.50%, viewers), and most were female (861,990/974,000, 88.50%). The gender breakdown was consistent through the series.

On a separate YouTube channel, the series provided episodes with French subtitles. Overall, the series with subtitles generated over 27,000 views and had a similar gender and age breakdown to the English series. Cote d’Ivoire was the most prevalent reported location of the audience (Table 2).

**Table 1:** Reported characteristics of *Alone Together* YouTube viewers: English channel (N=974,000).

Reported characteristics (English)			Viewers
**Gender, n (%)^a^**			
	Female	861,990 (88.50)	
	Male	112,010 (11.50)	
**Age group (years), n (%)^a^**			
	13-17	22,403 (2.30)	
	18-24	529,856 (54.40)	
	25-34	394,470 (40.50)	
	35-44	26,298 (2.70)	
	44+	974 (0.10)	
**Geographical location, n (%)**			
	Nigeria	336,030 (34.50)	
	Ghana	73,050 (7.50)	
	United States of America	61,362 (6.30)	
	United Kingdom	30,194 (3.10)	
	Canada	29,220 (3.00)	
	Kenya	13,636 (1.40)	
	South Africa	11,688 (1.20)	
	46 Other countries	36,038 (3.70)	
	Country unknown	382,782 (39.30)	

^a^Absolute number of viewers is estimated based on the total viewer population of 974,000 and percentage breakdown provided by YouTube Analytics (percentage of missing data were unavailable).

**Table 2:** Reported characteristics of *Alone Together* YouTube viewers: French channel (N=27,552).

Reported characteristics (French)		Viewers
**Gender, n (%)^a^**		
	Female	23,805 (86.40)
	Male	3747 (13.60)
**Age group (years), n (%)^a^**		
	13-17	0 (0)
	18-24	14,933 (54.20)
	25-34	12,619 (45.80)
	35-44	0 (0)
	44+	0 (0)
**Geographical location, n (%)**		
	Côte d'Ivoire	5621 (20.40)
	France	771 (2.80)
	États-Unis (United States of America)	110 (0.40)
	Italie (Italy)	110 (0.40)
	Maroc (Morocco)	55 (0.20)
	Sénégal (Senegal)	28 (0.10)
	Botswana	28 (0.10)
	18 other countries	165 (0.60)
	Country unknown	20,664 (75.00)

^a^Absolute number of viewers is estimated based on the total viewer population of 27,552 and percentage breakdown provided by YouTube Analytics (percentage of missing data were unavailable).

### Themes

#### Overview

There were 3985 comments posted in the YouTube comment section, and all 70 episodes had viewers engaging in live chat conversations. By examining reactions to *Alone Together* storylines and the opinions viewers expressed online, we identified the following perspectives on the COVID-19 pandemic and national lockdown measures expressed by the predominantly young female audience: (1) people have a social responsibility to follow COVID-19 measures; (2) lockdowns are creating significant economic challenges for people in their community; (3) social isolation is necessary but affects mental health; and (4) lockdowns make it difficult for people who experience intimate partner violence to seek help. We also identified ways in which watching the *Alone Together* series and engaging with MTV Shuga producers and fellow viewers online helped younger adults to cope with the pandemic by (1) forming online communities through shared experience; (2) asking for resources and information; (3) using the series as a link to the real world; and (4) enjoying entertainment together. We extracted comments to illustrate these themes and have presented them below. To accurately represent the viewers, we have presented comments using the spelling and slang that viewers use on YouTube.

#### People Have a Social Responsibility to Follow COVID-19 Measures

From the earliest episodes in April 2020, many commenters expressed that COVID-19 was serious (“COVID19 is definitely no joke”) and that everyone should follow public health measures to control the “virus” spread. “I just hope people take it seriously for the sake of the vulnerable amongst us.” Commenters reacted with anger when *Alone Together* characters chose to disregard social distancing rules. For example, they rebuked the character “Tobi” who planned an exclusive party during the countrywide lockdown in Nigeria.

This Tobi guy, he wants to infect about 100 people exclusively. It's alright. community service and fines day await.

[Reply] T you have no sense 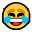

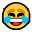

[Reply] Right? Like how dumb is that idea?' 'You'd think corona skips exclusive parties?

The audience reacted similarly to “Lemo” in South Africa when he chose to meet friends despite being exposed to his aunt, who had tested positive for COVID-19. “Bruh I’m this close to put my hands on that little boy Lemo like wtf 
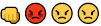
.” Commenters also criticized characters who did not wear masks or spread misinformation online and appealed to their online peers to keep themselves and others safe. They viewed individuals as selfish who were not taking COVID-19 seriously or did not recognize their role in protecting vulnerable people. This was most evident in response to the character “Joe Massive,” the Instagram influencer who contracts COVID-19 after encouraging his fans not to wear masks.

Hopefully Massive does another [Instagram] live video saying [COVID-19 is] real so that he can correct his wrong information. Not only positive vibes should be shared.

[Reply] Joe Massive is a wakeup call to everyone #Lets all stay safe.

[Reply] Massive please learn your lesson…. COVID is not a joke.

[Reply] Yes wear your mask please.

Many found these scenes relatable, and they mentioned people in their own lives who were not following social distancing measures.

Definitely going to parties and into large gatherings rn is not advised at all

[Reply] Right....my family just had a big wedding. I stayed home.

In the first 2 weeks of the show, there were a few comments in which viewers were distrustful or skeptical with COVID-19 health messages around lockdowns and social distancing.

No offence it’s two COVID cases in Nigeria, why y’all in lockdown?

The propagandists have polluted the whole world with this economy killing lockdown BS […] this sh*t is toxic and detrimental to black folks. unsubscribed.

Although many viewers reacted very strongly to characters who were not adhering to COVID-19 regulations, viewers did not engage with comments from their peers online who resisted COVID-19 measures. Comments opposing COVID-19 measures became less frequent as the series progressed.

#### Lockdowns Are Creating Significant Economic Challenges for People in Their Community

Many episodes prompted viewers to comment on the economic hardship that COVID-19 mitigation measures created for many people.

Covid 19 is definitely not a holiday, it’s really money sapping and the lockdown is limiting.

Commenters reminded themselves and their online peers to be compassionate and grateful.

I love how @MTV Shuga brought the issue of ‘hunger virus’, people are literally going through hell in times like this. A little kindness will save a life.

For example, in one episode, the character “Leo” complains about being bored in lockdown. His friend “Zamo” tells him he is being selfish and is lucky to live in a comfortable, luxurious home, and commenters agree.

Leo was a bit selfish.

[Reply] Zamo gonna be real with him.

[Reply] He is maybe a little bit…he should be supportive [to Zamo].

One commenter reinforces this message of awareness and gratitude by openly reflecting on their privilege of access to the internet and electricity, which her online peers, who can watch *Alone Together* online also share.

I remember chatting with my brother in Nigeria that I am bored and he said I have electricity, internet and food, why was I complaining. I felt bad 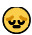

Audiences reacted to the storylines of “Wasiu” (based in Lagos) and “Zamo” (in Johannesburg), who were both without income during lockdowns in Nigeria and South Africa, by discussing how to navigate a challenging economic situation during the pandemic. Commenters acknowledged the difficulty of finding work during the lockdown or making money safely and responsibly. At a point in the series when “Wasiu” seeks to supplement his rent by letting strangers with possible COVID-19 symptoms sleep in his home, commenters weighed the risk in his decision as they discussed his potential exposure to COVID-19 versus his economic situation.

Wasiu shouldn’t have allowed [people to live with him].

[Reply] But he needs the money.

[Reply] He needs to find a job, a real one.

[Reply] Wasiu has collected the money for subletting so he either returns the money or he deals with the consequences.

[Reply] Where is the job in this lockdown?

In this exchange, 1 commenter links this scenario to the real world, noting the scarcity of work many people face during the lockdown. Commenters demonstrated shared creative ways for the characters to make money within the constraints of lockdown, possibly providing advice to their peers who are struggling in real life.

Zamo can give beauty tutorials classes online and charge a registration fee. She can make money that way and not have to leave her house, she can even link up with Leo to help her promote it.

#### Social Isolation Is Necessary but Affects Mental Health

Social isolation was another topic in the series that resonated strongly with commenters: “Truth is we need human interaction everyday.” Many were sympathetic to the series’ characters “Leo” and “Daniel” who lived alone during the lockdowns in Kenya and Cote d’Ivoire, respectively.

Daniel doesn’t look ok at all.

[Reply] He definitely doesn’t. Tough time to be living on one’s own, away from family.

Commenters explained how loneliness and isolation impacted their mental health. They described being stuck at home alone as depressing, unnaturally sad, draining, limiting, and boring.

Leo, it always depressed- but I understand that being stuck in the house 24/7 isn’t easy.

[Reply] Yes it isn’t easy.

[Reply] It can drain you mentally.

[Reply] Leo it’s ok not to be ok.

They also explained how being unable to control their loneliness due to social distancing measures and lockdowns worsened their situation.

Let us not forget persons living on their own (from the young adults to the elderly). It’s one thing to be alone, but to be lonely and not be able to do much about it, is sad.

One viewer sympathized with the character Mbali, whose mental health is suffering while isolating due to a COVID-19–positive test. She comments that Mbali should go see a friend because of the toll isolation is taking on her mental health. Other viewers responded that Mbali should continue her isolation.

Mbali looks a mess, Jesus. She should go hang out if it’ll make her feel better, lol.

[Reply] No, I think she should just avoid the risk and stay safe at home.

[Reply] Personally, I don’t think she should.

[Reply] She definitely needs to isolate.

[Reply] Mbali should not go, I sense danger.

[Reply] Mbali looks fine - She just needs to heal.

Commenters expressed the challenges for those who are away from their family at this time. “Being without my family all this time is draining.” One viewer shared their painful experience of losing their father and being unable to travel to attend his funeral. Online peers offered support, empathy, and condolences.

I lost my dad this period and they buried him, I can’t even go and pay the last respect… I’m so much in pain 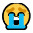

[Reply] So so sorry for your loss. I believe he’s resting.

[Reply] May his soul rest in peace amen.

[Reply] Hard luck dear, it is so painful.

[Reply] Ooh sorry dear...may the good Lord comfort you.

#### Lockdowns Make It Difficult for People Who Experience Intimate Partner Violence to Seek Help

Many commenters appreciated a storyline about partner violence as they felt it reflected current reality, especially during the pandemic.

My heart breaks for the women who actually go through this especially now during the lockdown. My mom left my abusive father this year and my heart is at peace finally cause I know the pain she felt.

The dramatic arc in the storyline for the character “Dineo”—in which it becomes evident that she is living with a violent partner during the lockdown—generated many responses and discussion. Although some commenters blamed Dineo for her situation, “I don’t feel sorry for her because when a relationship is toxic u should leave,” online peers explained why it was difficult for women to leave violent relationships, even more so during a pandemic.

Dineo can’t just leave during this pandemic if she got no place to go.

Look at [Dineo’s] situation. She can’t leave even to go buy something, he won’t let her. The most she can do is call the police or a hotline but she isn’t even mentally capable cause he is fooling and abusing her.

I've heard experiences of ladies in Dineo's shoes! Once you're in a relationship with a manipulative abuser, it's hard to leave them.

Commenters agreed that despite the COVID-19 lockdown measures, it was important that Dineo finds a safe way to leave home and escape the violence.

Dineo get out! Pandemic or not this situation isn't good for your physical or mental health! Talk to someone anyone!

Most comments on episodes about intimate partner violence were oversimplified calls for Dineo to leave her relationship “Dineo, run”. Only a few viewers offered strategies for leaving the violent relationship.

Dineo.....gurrrrll you better order a pizza. 911 child!

This having a code word [to signify to a friend you are in trouble] is definitely a great thing.

People! Know where your friends live! Code word for what if you can't find them when they're in trouble. Sha!

#### Creating an Online Community Through Shared Experience

The comment section reflected a desire from viewers to connect with their peers by sharing advice, support, and their own experiences during the pandemic. Conversations on the *Alone Together* online platform offered a socially distant way to reactivate community engagement, while in-person connections were restricted. The shared experiences and hardships created by the COVID-19 pandemic, enacted by *Alone Together* characters, helped develop a sense of community. One common way viewers utilized the comments was to thank frontline workers.

Shout out to all health workers out there.

Salute to all our frontline health workers 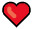

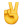

They also urged their peers to think communally and raised awareness about those making sacrifices or struggling during the pandemic.

I appreciate the introduction of the hospital cleaners and other non-medic staff in hospitals as high-risk workers. We should remember them as we pray and celebrate our doctors, nurses and lab technicians etc.

Prayer for single mothers out there 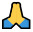 through this pandemic 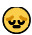

Viewers also used the online community to build camaraderie with other viewers and encourage optimism and resolve despite the challenges of the pandemic and lockdowns.

Wait, am I the only one seeing this loneliness brings love? I guess there's something positive about COVID-19.

[Reply] True.

[Reply] Yh, like you have time for others.

They expressed how their engagement with *Alone Together* went beyond watching the series and helped to connect them across countries and cultures.

Been watchin Shuga since day one from a French country, first time to comment but I just wanted to say this is a real family. From different places but a real family 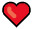

#### A Link to Resources From *Alone Together*

*Alone Together* raised awareness about available mental health resources, abortion care, domestic violence, and COVID-19. Viewers used the comment section to connect directly with the *Alone Together* team to receive links to resources that fit their individual need.

I actually need these links to talk to these mental health organisations, MTV Shuga if it's possible please.

[Reply] MTVShuga: "Hi, please let us know what country you're in at the moment, or visit www.mtvshugaalonetogether.com to access the helplines for the countries currently available. Thank you.

Viewers also used the comment section to ask *Alone Together* producers for more education and awareness about specific scenarios that people might be facing during COVID-19. Some of the requests included characters (some from past series of MTV Shuga) struggling with family support, pregnancy, and addiction.

@MTVShuga you should also address addiction, especially now.

@MTVShuga, I want to see [the character from MTV Shuga, Down South] Sol, a lot going there, losing his mum, taking care of his sis and then Covid-19 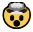... a lot.

#### Used the Series as a Link to the “Real World”

Commenters expressed that they found the series realistic and reflected on what is happening in real life. As viewers grappled with isolation and disconnection from their communities, *Alone Together* served as a link to unite viewers to fictional but relatable community members. “This is like a glimpse into the outside world...” Watching the series raised their awareness of how COVID-19 might be affecting certain groups disproportionately:

Hmmm cannot imagine being a child and having to deal with a sick parent or guardian with a contagious disease. Great job again MTV Shuga.

Single mothers must be going through alot RN.

[Reply] Honestly they are...I wonder how they are coping?

[Reply] A day at a time is what I am doing.

Commenters were empathetic toward characters who were sick with COVID-19 or had a loved one who was ill. Additionally, the series made them aware of the working conditions in overwhelmed hospitals and the sacrifices frontline workers made for their community.

I legit started crying when [the character] Dr. Sope started talking about corona virus patients.

[Reply] It takes a lot of gut to be a nurse or a doctor.

#### Enjoying Entertainment Together

People tuned into the series as a source of support during the pandemic. Commenters applauded the episodes’ short and daily format, which served as a consistent and enjoyable moment in their day during an unstable time. For some people, watching *Alone Together* “kept them sane” and lifted their mood:

Love love this show... It's amazing how I look forward to my 5 exciting minutes 5 days a week… hum not bad huh? Looking forward to Monday 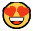

On the season finale, commenters shared that tuning into the series made lockdown more enjoyable and expressed that the series had been a source of comfort to them over the past months.

I appreciated what you guys did as a way to occupy our minds and attention during the pandemic. All in all I want to say thank you for #alonetogether it was entertaining for me as well as being informative #alonetogether #staymasked #covidisreal #SENDINGLOVEFROMTHEBAHAMAS

Am so proud of you all for putting all this beautiful content together through this hard, scary and stressful moment. Love all your content. ALONE TOGETHER 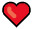. All this will be something of the past by God's grace.

Thank you so much MTV Shuga for keeping me informed and entertained, you guys make me wanna do better and I’m going to miss 'Alone together'.

## Discussion

### Principal Findings

Our findings showed that the new miniseries based on COVID-19—*MTV Shuga: Alone Together*—reached dominantly female audiences who understood the importance of individual behaviors to prevent COVID-19, and encouraged their online peers to follow COVID-19 mitigation measures. However, the audience also empathized and related to those suffering the most from COVID-19 lockdowns and were significantly concerned about the psychosocial and economic implications of strict COVID-19 mitigation measures. Thus, there appeared to be a struggle between social responsibility (following COVID-19 prevention rules for the social good) and the isolation and hardship that this entailed for people. In the absence of face-to-face meet-ups due to social distancing measures, *Alone Together*’s YouTube page became a place where people could safely connect and discuss current events and their personal experiences. It became a valued support system, particularly for adult women under 35 years of age, during the first wave of the COVID-19 pandemic. Viewers used the *Alone Together* social media platform to share support, decision making, and coping strategies to face challenges created by the pandemic measures, uniting viewers across diverse places and cultures.

*Alone Together* reached at least 53 countries, with the largest reported audience in Nigeria, where 4 previous seasons of the MTV Shuga series were filmed and have an established MTV Shuga fanbase. The United States, UK, and Canada also had high reported viewership, which is likely aided by very high-speed internet access in these countries. Episodes with French subtitles had the most reported viewers in Cote d’Ivoire, which is the location for the latest series of MTV Shuga. Although MTV Shuga creates its content for young people (ages 15-24 years), 43.37% (434,361/1,001,552 viewers) of French and English viewers were above the age of 24 years. MTV Shuga also aims to develop gender-balanced content; however, overall, the MTV Shuga series are more popular with a female audience on YouTube. *Alone Together*, which likely gathered viewers from fans who had watched the previous series of MTV Shuga, was more successful at attracting female viewers from the premiering episode. Young men appear less receptive to watching or are unaware of the MTV Shuga series on YouTube. Although men in the analysis are unrepresented, it allows us to understand the views, perceptions, and experiences of women under the age of 35 years, primarily in countries across Africa and the United States, Canada, and UK, during the pandemic.

The social and economic challenges of the COVID-19 pandemic have had an immense impact on the daily lives of younger adults. Viewers shared how national lockdowns had exposed themselves and people they knew to economic uncertainty. People in their 20s and 30s are more likely than other age groups to work in sectors heavily affected by the pandemic. In particular, women were more likely to lose employment and take on unpaid care responsibilities [10]. Oxfam International estimated that COVID-19 has cost women US $800 billion in lost income globally in 2020 and has widened both gender and economic inequalities [11,12]. Viewers also discussed their isolation and loneliness and being away from family and friends during this time. A UK study showed that adults aged 18-30 years and adults living alone experienced a greater risk of loneliness during the COVID-19 pandemic than other groups [13]. Young people with existing mental health illnesses have reported that COVID-19 made their condition worse, and for some, it restricted their access to mental health services [14]. Additionally, the national lockdowns across the world caused spikes in household violence, leaving girls and women particularly vulnerable [15]. Viewers struggled to find solutions for the character in the series that was stuck in a violent relationship. This issue played out in real life as women worldwide reported difficulties accessing essential gender-based violence services because they could not leave their homes or seek help online or over the phone [14,16].

Social media provided viewers with a space to raise awareness about the challenges they face during the pandemic and ask for the resources they need. For many younger adults, online connections are as much a part of their social life as in-person peers and have possibly become more vital during lockdowns and social distancing. If channeled in positive and constructive ways, social connection online can buffer anxiety and depression induced by isolation in the pandemic and offer support in the absence of in-person contact [17]. Social media can also be a useful public health tool for establishing prosocial public health behaviors. Content on social media influences people’s perception of their peers’ behavior, affecting their own intentions for that behavior [18]. Young people are especially susceptible to peer influence and have a great capacity to influence one another [19]. Viewers who engaged with or consumed *Alone Together*’s social media content saw their online peers imploring others to follow COVID-19 measures despite the psychosocial impact and to show empathy, compassion, and concern for people struggling during the pandemic. Commenters mainly were united in these expressions, establishing social norms about the appropriate and ethical behavior their peers should demonstrate during the first waves of the COVID-19 pandemic.

### Limitations and Strengths

Those who watched *Alone Together* represent individuals with access to media (the internet and a device). Their experiences of COVID-19 may be different from those who lack such access. This audience is more likely to have access to other resources and may experience fewer challenges due to COVID-19. In such situations, the choice to act with social responsibility may come with fewer personal consequences (eg, than somebody who is facing hunger and homelessness). Furthermore, those who commented on the series comprised only a small percentage of viewers and may not represent all viewers. Demographic information extracted by YouTube Analytics represents all viewers, not just those who post comments. YouTube analytic data are self-reported, so viewers can select their gender and age. Young people under 18 years might be motivated to set their age to over 18, so their content is not restricted. A large number of participants chose not to share their location information with YouTube, causing the location data to be incomplete. Additionally, by excluding non-English comments, the voices and experiences of viewers from French-speaking countries will not be represented in the data. Viewers were free to choose if and how they engaged in topics, so commenters may reflect viewers who are more confident, experienced (with digital media), have better internet access, or are more engaged and supportive of the show. This may explain in part why most comments were positive and supportive, and relatively few commenters challenged the content or themes raised in the series. Also, those who were not receptive to *Alone Together*’s messages could have unsubscribed, stopped watching the series, or stopped posting comments.

Although social media’s anonymous nature can enable candid, honest comments and viewpoints, it is also possible that viewers can misrepresent themselves [20]. For example, in their YouTube account, viewers could list themselves as a different age or gender. Their comments could present a persona or opinions that differ from those they offer in person. By contrast, viewers who are truthful about their identity could have potentially shied away from sharing opinions, personal experiences, and asking for help due to fears of judgment, identification, or cyberbullying. These factors could influence the comments’ validity and the risk of treating social media posts as empirical data.

Despite these limitations, most comments were validated through the agreement of fellow viewers, and it was clear (from responses to posts) that viewers found value and truth in what their peers posted. There was little reason or incentive for viewers to misrepresent themselves, and we have assumed that most are sharing honest views. The comments represent viewers’ own choices about what to share, and thus reflect the themes and experiences that resonated most for them. Our study result offers a valuable first-person perspective from young people facing a dramatic global event. As future waves of COVID-19 infections and lockdown measures occur, their reactions provide insights and lessons to help support young people through further challenges.

### Conclusions

This analysis has shown how younger adults across the globe responded to the first wave of the COVID-19 pandemic and the strict lockdowns implemented in most countries. These viewers—predominantly women under 35 years of age who were digitally connected and engaged—recognized the critical role they played in keeping other people safe, even at the expense of their own economic and social needs. They recognized the immediate and lasting impact that COVID-19 restrictions were having on younger adults’ mental health, livelihoods, and relationships. As future waves of infection and lockdown occur, especially in low- and middle-income countries with limited COVID-19 vaccine availability, it will be increasingly difficult for younger adults to continue to make such sacrifices. Support systems are needed, and within those edutainment supported by social media can offer connectedness and linkages to resources to cope with the COVID-19 crisis. Viewers who commented on the series showed that they are receptive to receiving support from online communities and media services. Online, people have a great capacity to influence each other and establish social norms. Peer influence and support online can be a powerful public health tool, particularly during a pandemic where individual behaviors need to shift rapidly to prevent disease transmission.

## Appendix

Multimedia Appendix 1 Final Digital Report of <italic>MTV Shuga: Alone Together</italic> 16 April - 25 September, 2020.

## Abbreviations

MTV SAF: The MTV Staying Alive Foundation
