# Supplementary material for: Young Adults’ Responses to an African and US-Based COVID-19 Edutainment Miniseries: Real-Time Qualitative Analysis of Online Social Media Engagement
Source: JMIR Form Res. 2021 Oct 29;5(10):e30449. doi: 10.2196/30449 (PMC8562417; doi:10.2196/30449)
Supplement: Multimedia Appendix 1 [file formative_v5i10e30449_app1.pptx]

## Slide 1
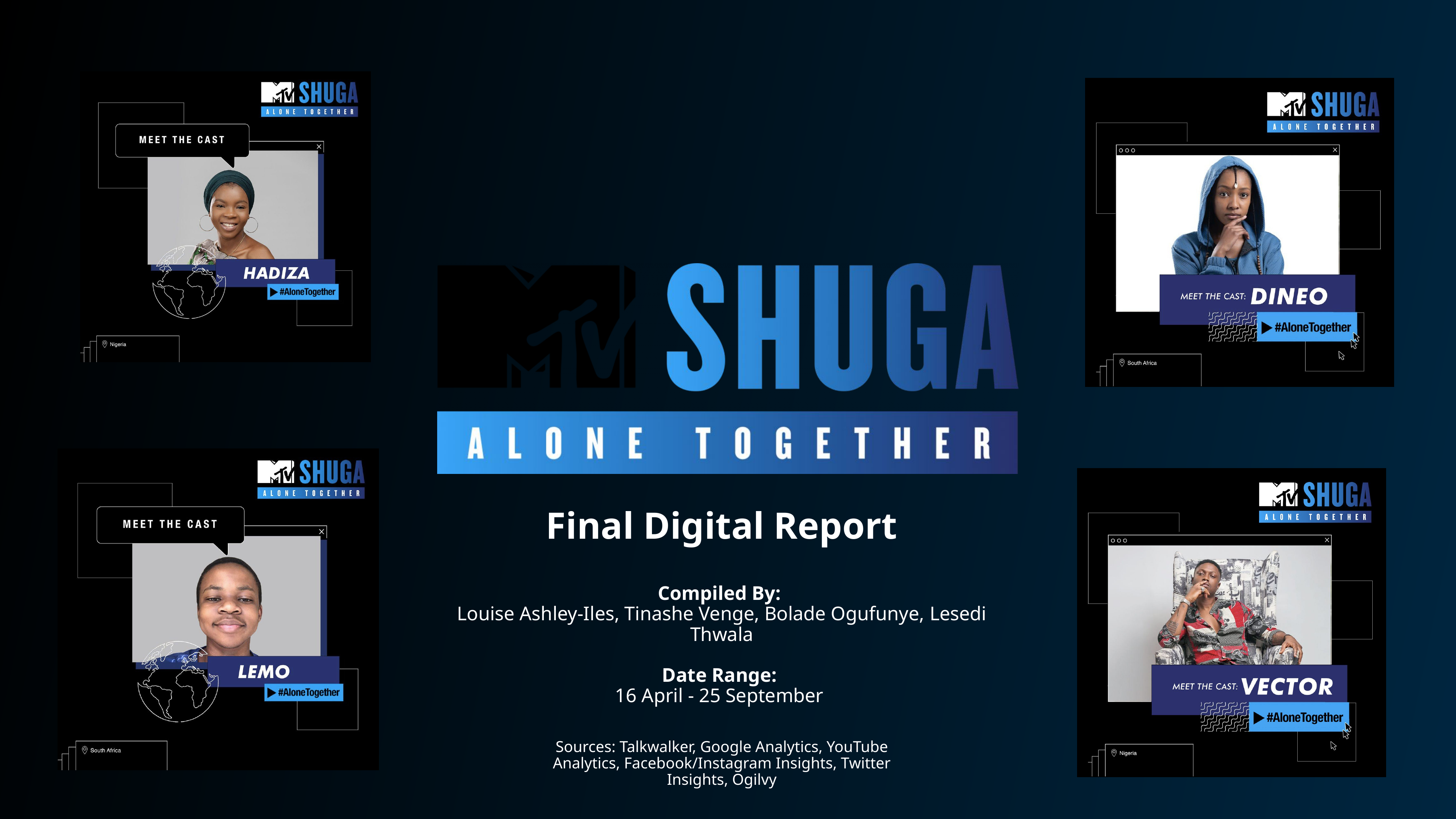

Final Digital Report
Compiled By:
Louise Ashley-Iles, Tinashe Venge, Bolade Ogufunye, Lesedi Thwala
Date Range:
16 April - 25 September
Sources: Talkwalker, Google Analytics, YouTube Analytics, Facebook/Instagram Insights, Twitter Insights, Ogilvy

## Slide 2
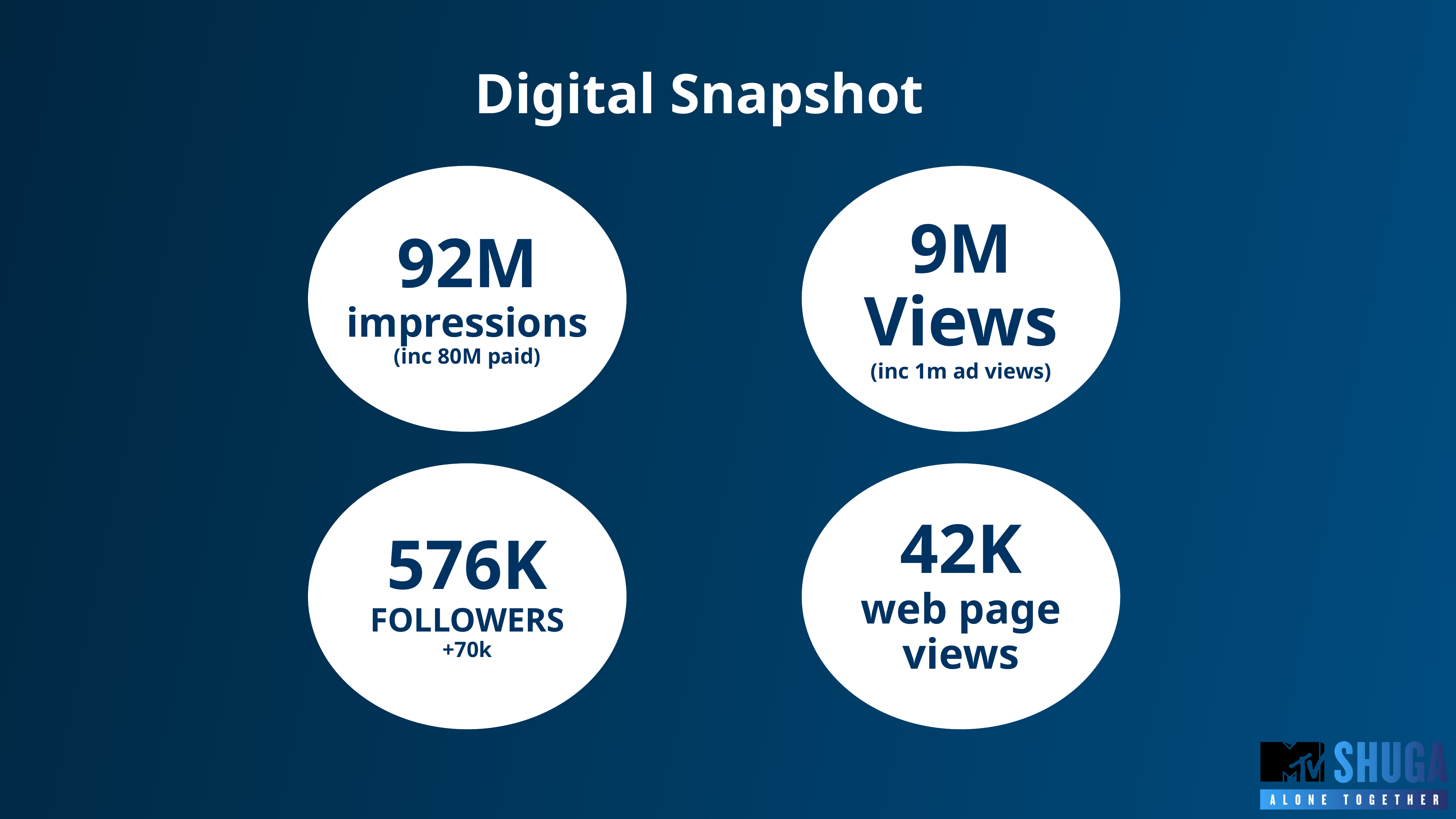

Digital Snapshot
92M
impressions
(inc 80M paid)
9M Views
(inc 1m ad views)
576K FOLLOWERS
+70k
42K web page views

## Slide 3
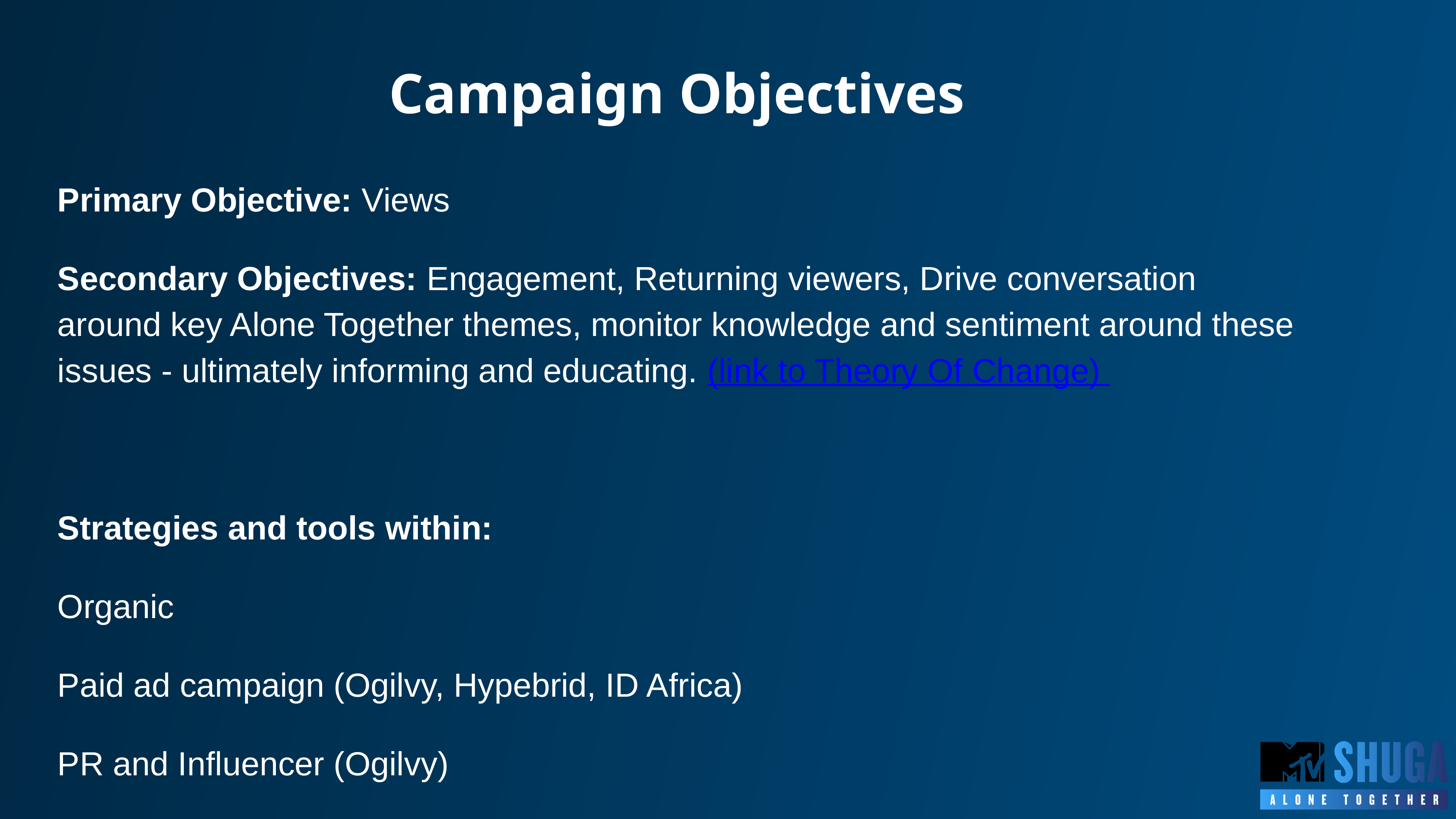

Campaign Objectives
Primary Objective: Views
Secondary Objectives: Engagement, Returning viewers, Drive conversation around key Alone Together themes, monitor knowledge and sentiment around these issues - ultimately informing and educating. (link to Theory Of Change)
Strategies and tools within:
Organic
Paid ad campaign (Ogilvy, Hypebrid, ID Africa)
PR and Influencer (Ogilvy)

## Slide 4
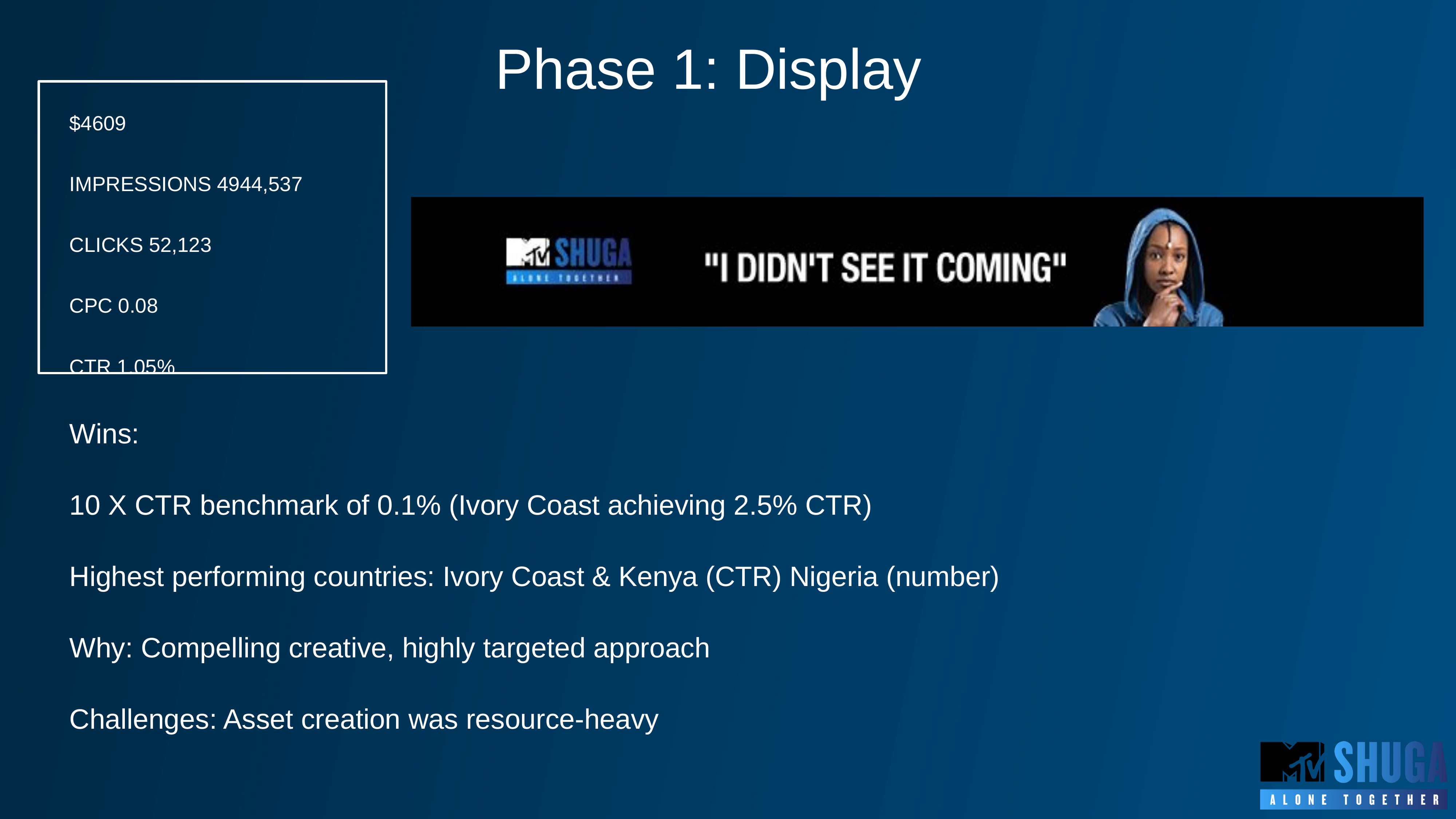

Phase 1: Display
$4609
IMPRESSIONS 4944,537
CLICKS 52,123
CPC 0.08
CTR 1.05%
Wins:
10 X CTR benchmark of 0.1% (Ivory Coast achieving 2.5% CTR)
Highest performing countries: Ivory Coast & Kenya (CTR) Nigeria (number)
Why: Compelling creative, highly targeted approach
Challenges: Asset creation was resource-heavy

## Slide 5
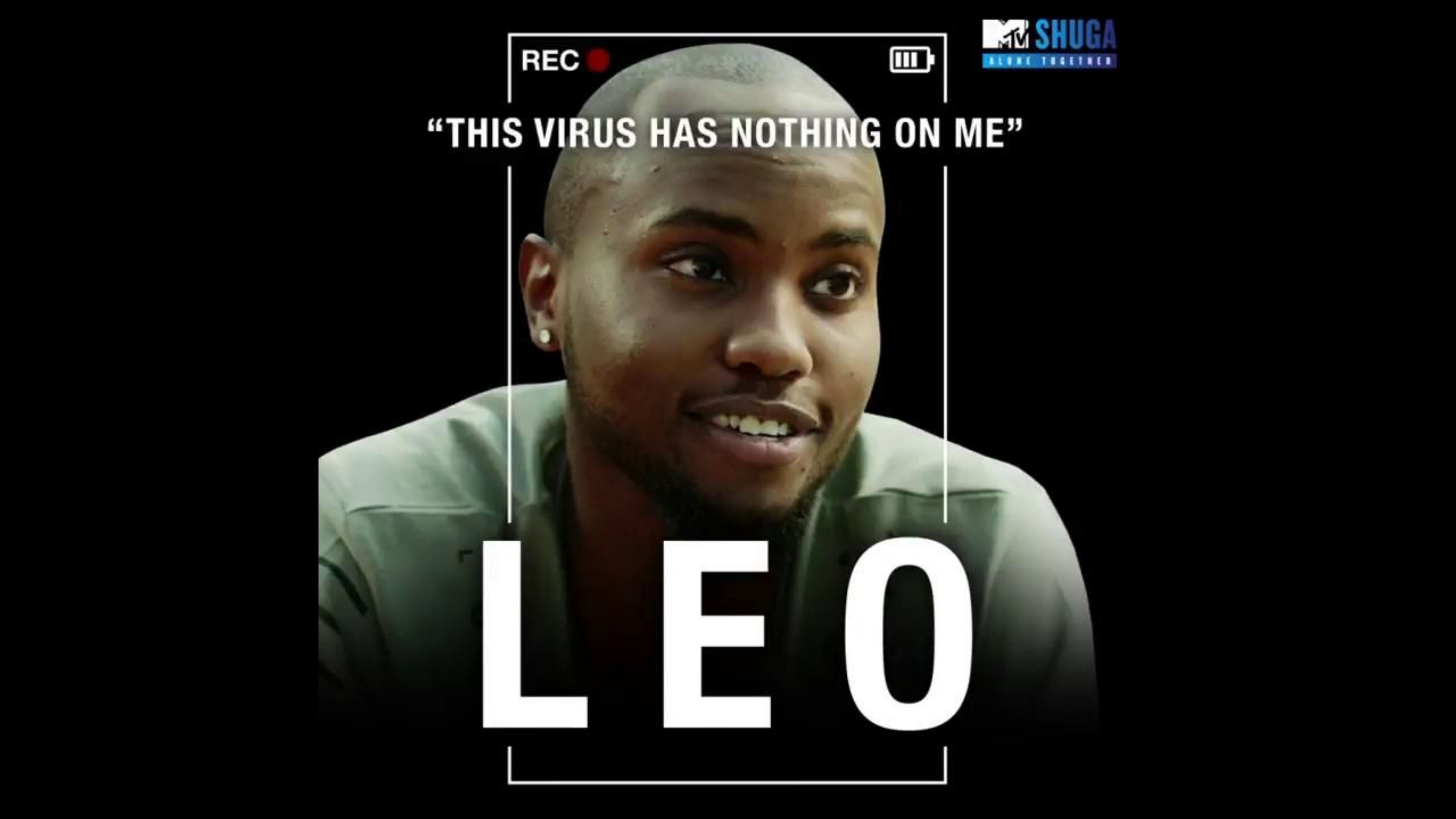

#

## Slide 6
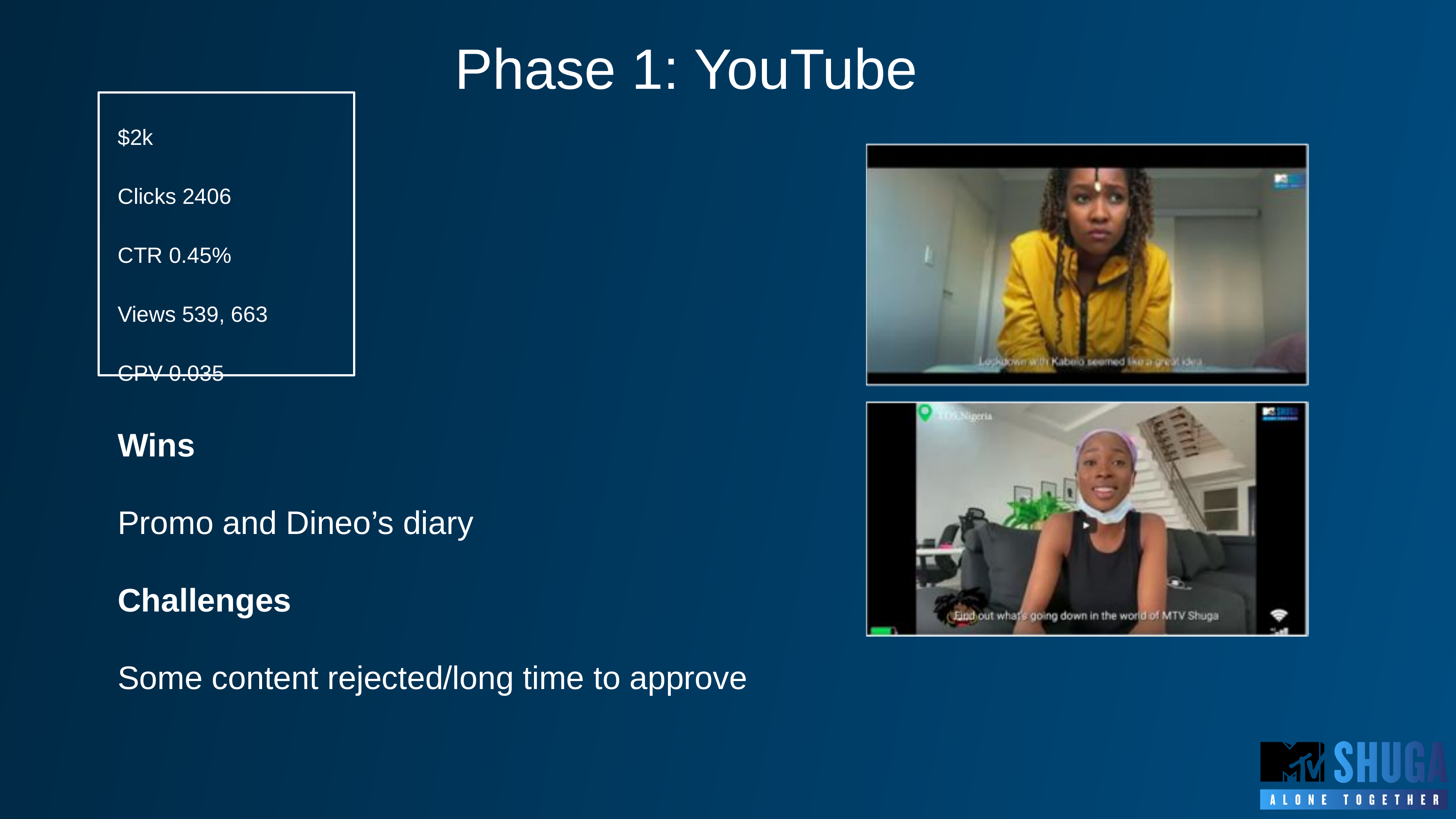

Phase 1: YouTube
$2k
Clicks 2406
CTR 0.45%
Views 539, 663
CPV 0.035
Wins
Promo and Dineo’s diary
Challenges
Some content rejected/long time to approve

## Slide 7
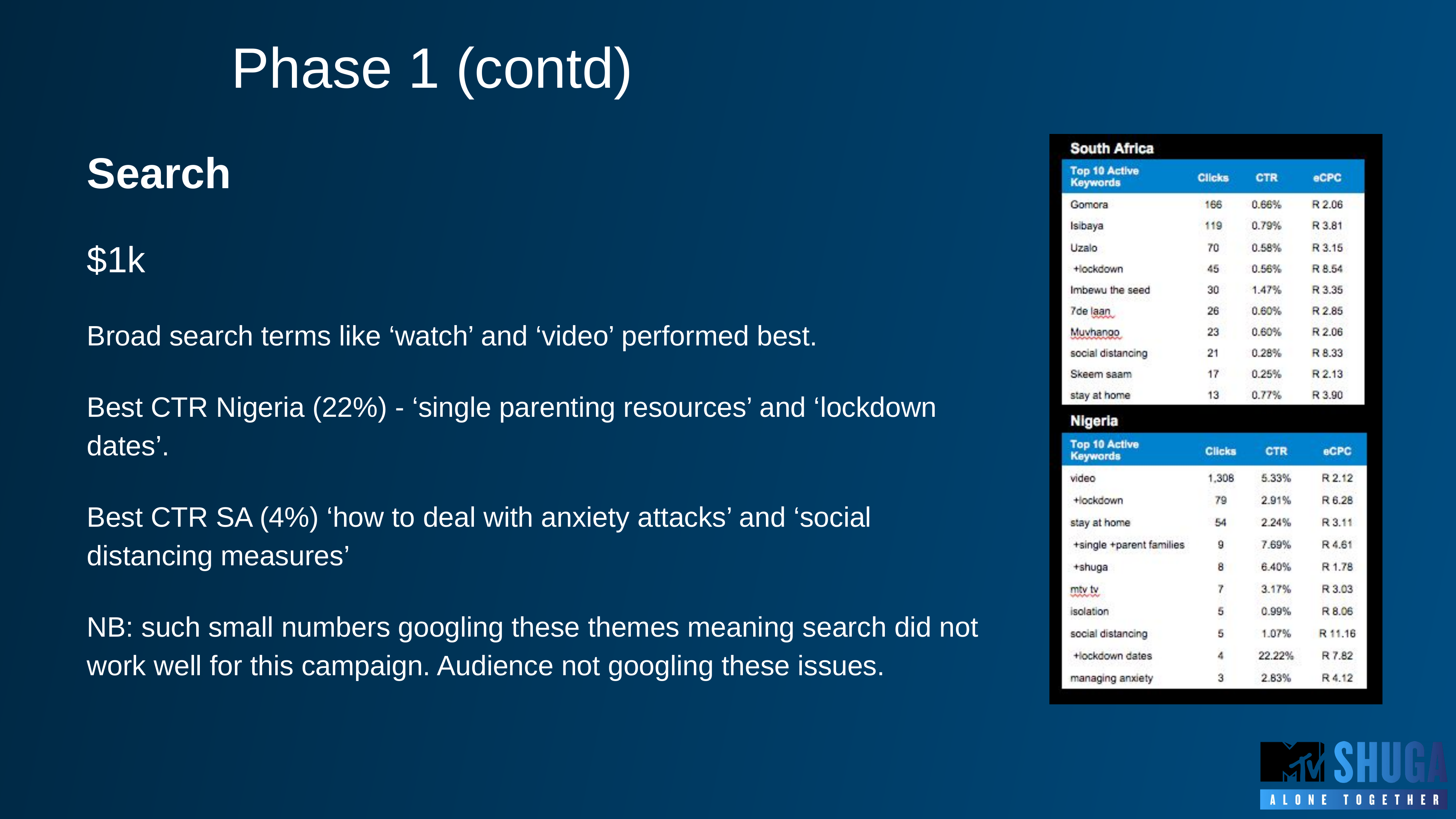

Phase 1 (contd)
Search
$1k
Broad search terms like ‘watch’ and ‘video’ performed best.
Best CTR Nigeria (22%) - ‘single parenting resources’ and ‘lockdown dates’.
Best CTR SA (4%) ‘how to deal with anxiety attacks’ and ‘social distancing measures’
NB: such small numbers googling these themes meaning search did not work well for this campaign. Audience not googling these issues.

## Slide 8
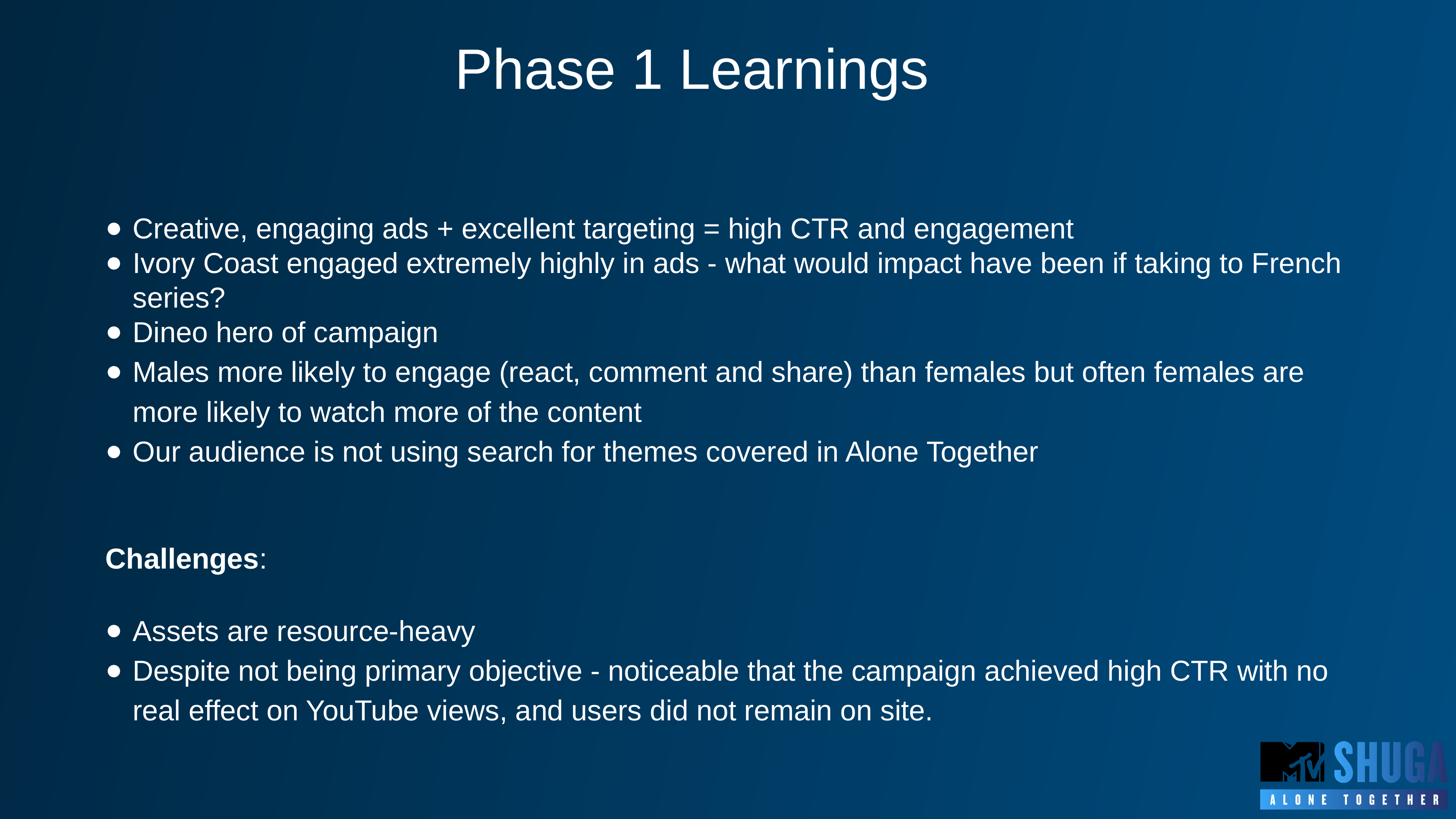

Phase 1 Learnings
Creative, engaging ads + excellent targeting = high CTR and engagement
Ivory Coast engaged extremely highly in ads - what would impact have been if taking to French series?
Dineo hero of campaign
Males more likely to engage (react, comment and share) than females but often females are more likely to watch more of the content
Our audience is not using search for themes covered in Alone Together
Challenges:
Assets are resource-heavy
Despite not being primary objective - noticeable that the campaign achieved high CTR with no real effect on YouTube views, and users did not remain on site.

## Slide 9
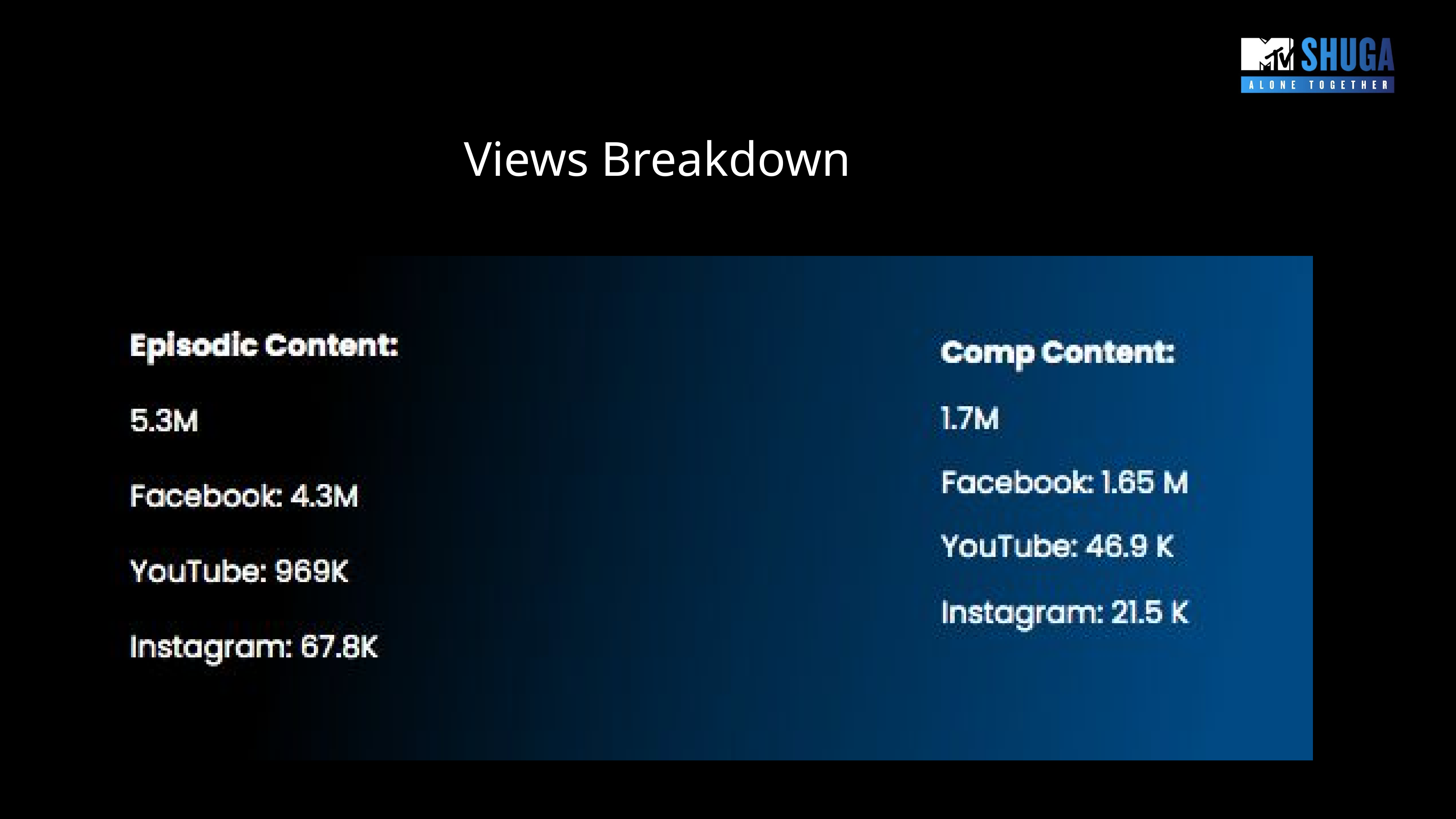

Views Breakdown

## Slide 10
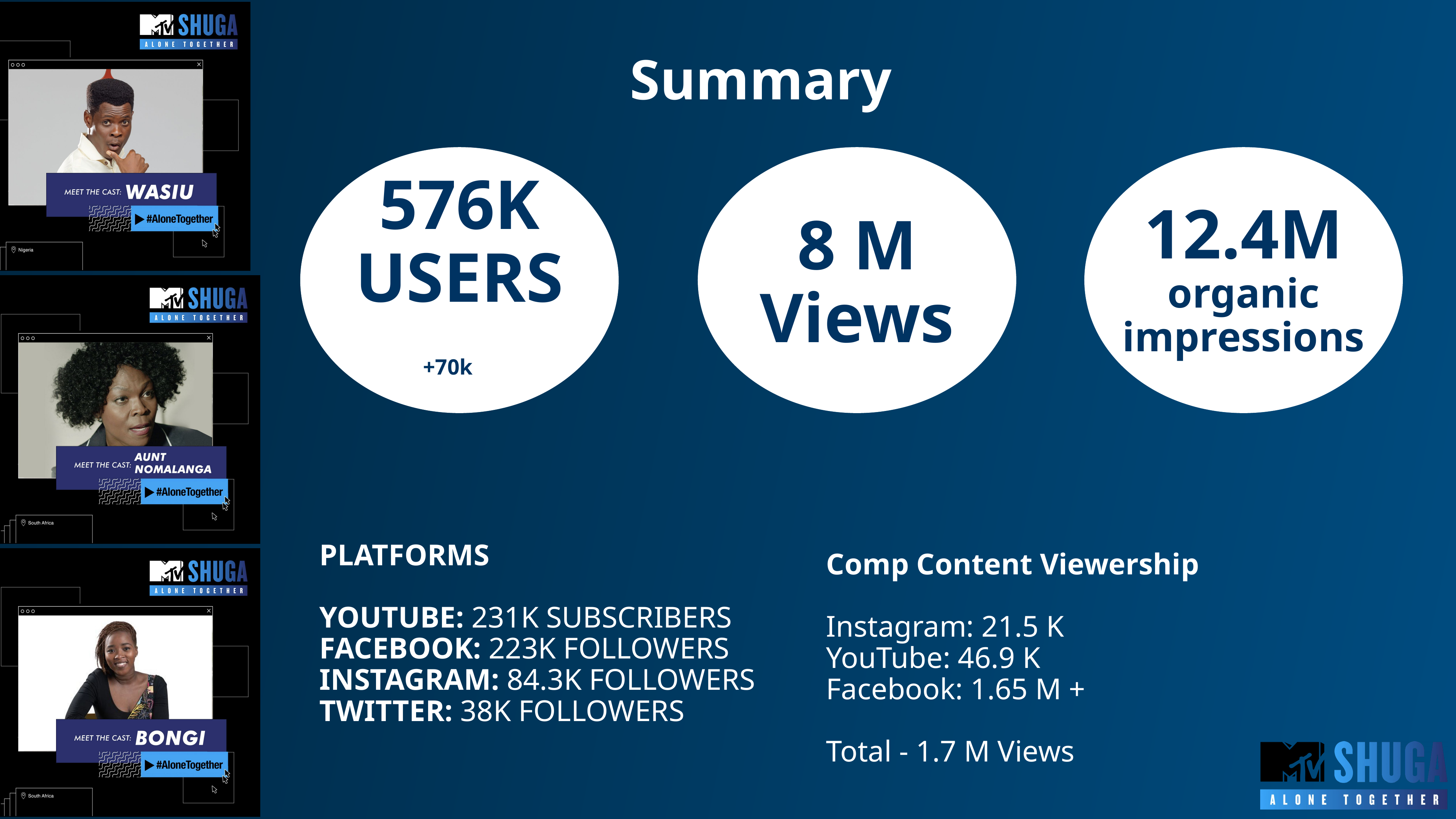

Summary
12.4M organic impressions
8 M Views
576K USERS
+70k
PLATFORMS
YOUTUBE: 231K SUBSCRIBERS
FACEBOOK: 223K FOLLOWERS
INSTAGRAM: 84.3K FOLLOWERS
TWITTER: 38K FOLLOWERS
Comp Content Viewership
Instagram: 21.5 K
YouTube: 46.9 K
Facebook: 1.65 M +
Total - 1.7 M Views

## Slide 11
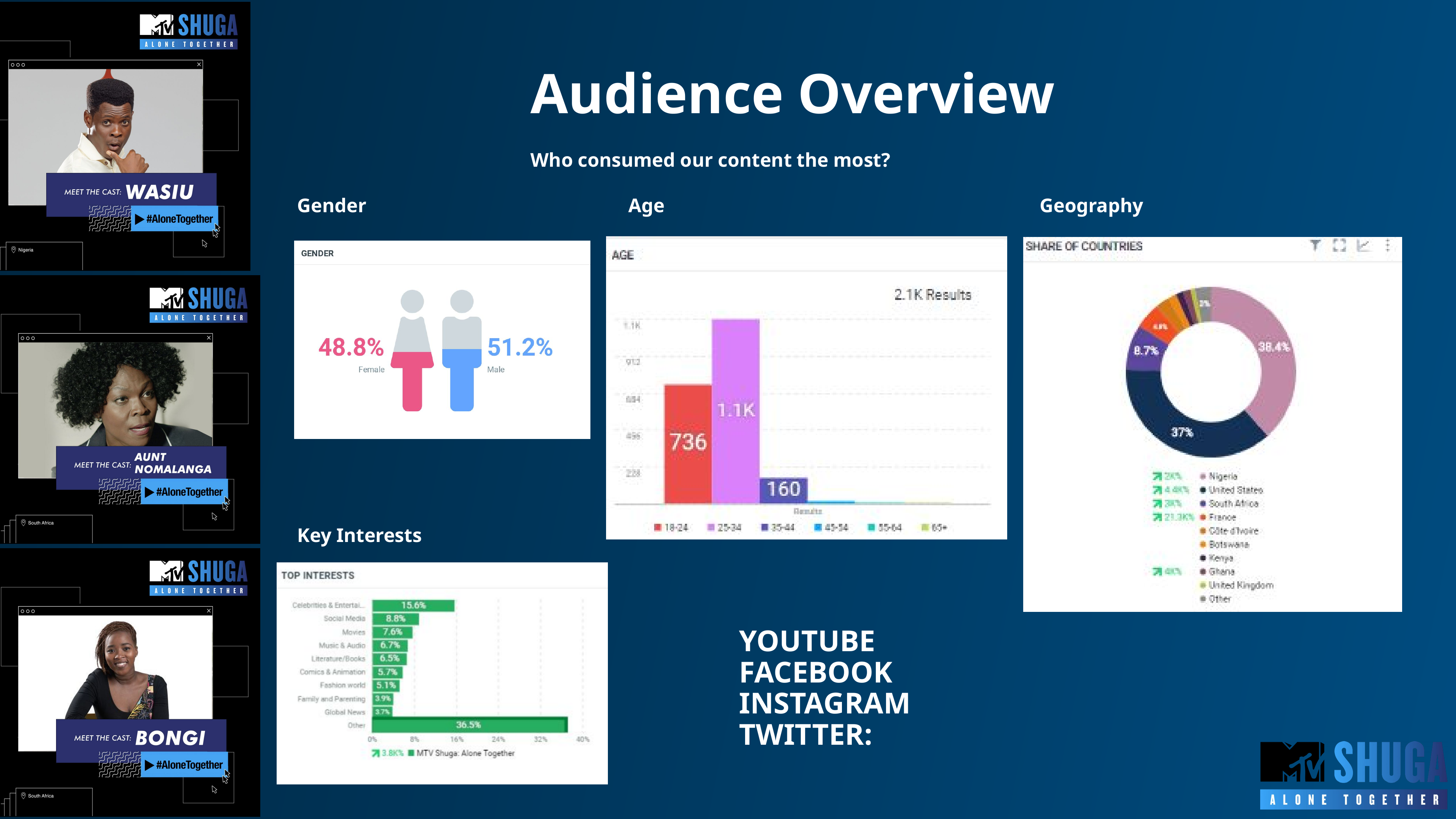

Audience Overview
Who consumed our content the most?
Gender
Age
Geography
Key Interests
YOUTUBE
FACEBOOK
INSTAGRAM
TWITTER:

## Slide 12
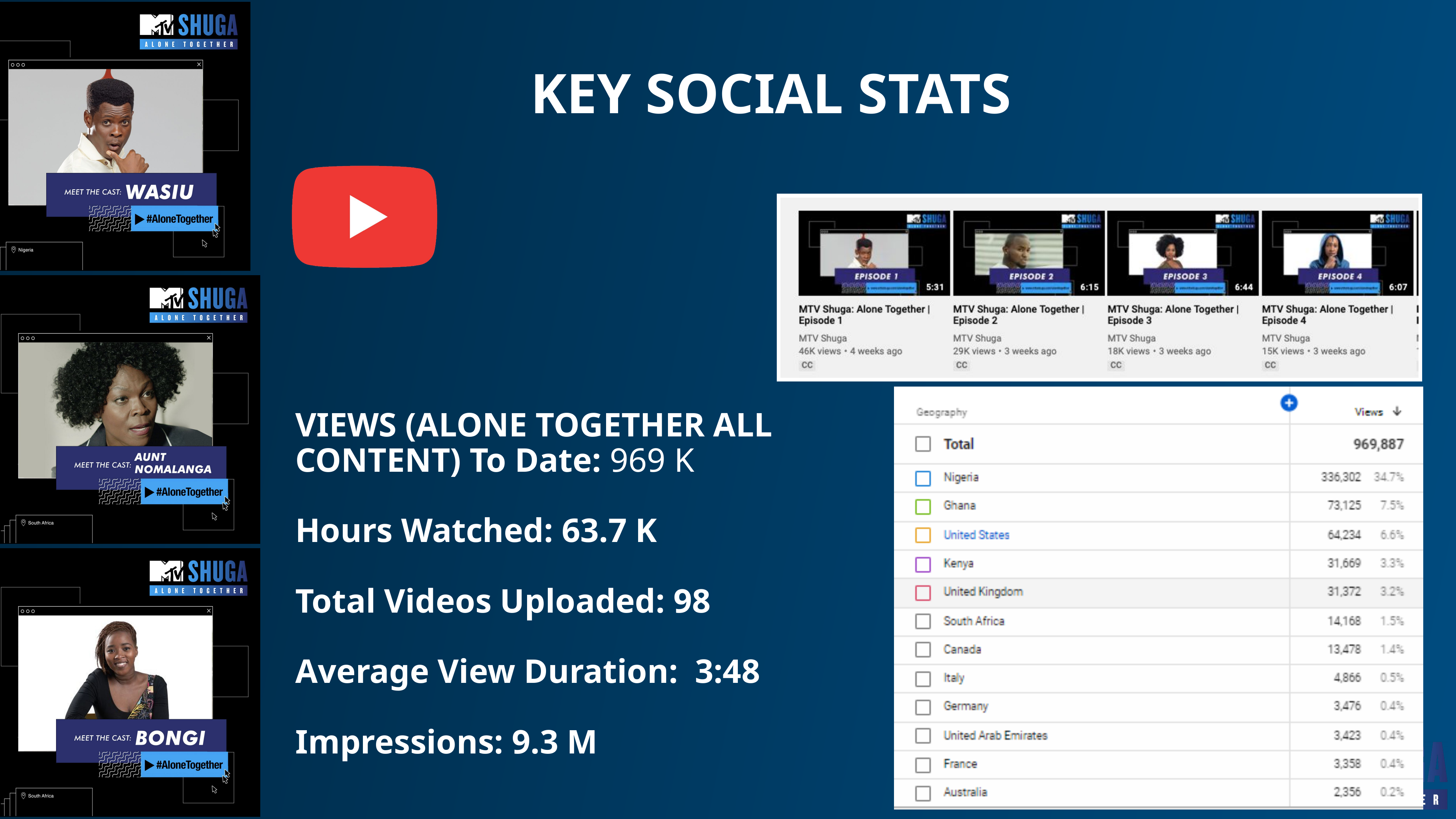

KEY SOCIAL STATS
VIEWS (ALONE TOGETHER ALL CONTENT) To Date: 969 K
Hours Watched: 63.7 K
Total Videos Uploaded: 98
Average View Duration: 3:48
Impressions: 9.3 M

## Slide 13
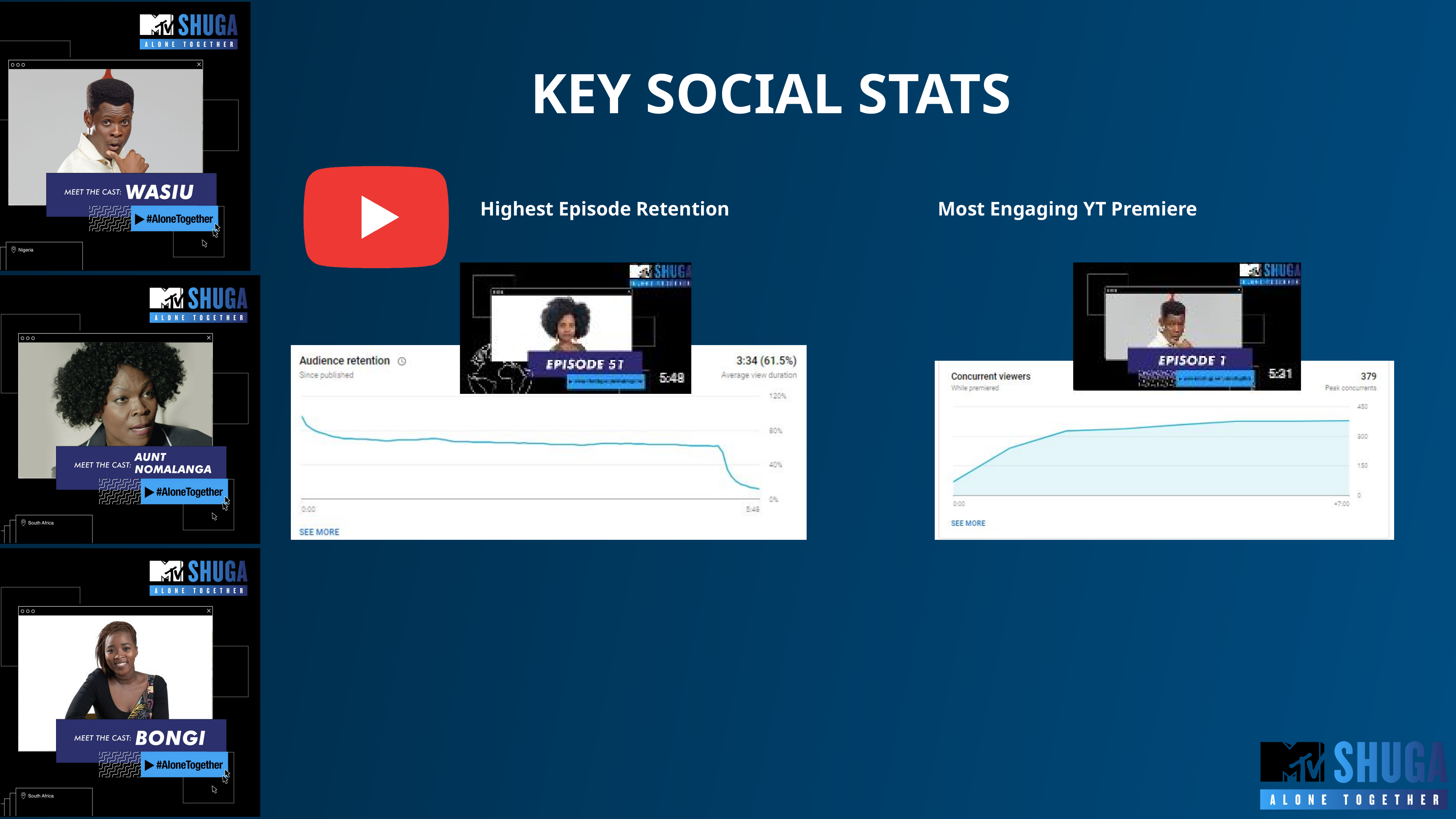

KEY SOCIAL STATS
Most Engaging YT Premiere
Highest Episode Retention

## Slide 14
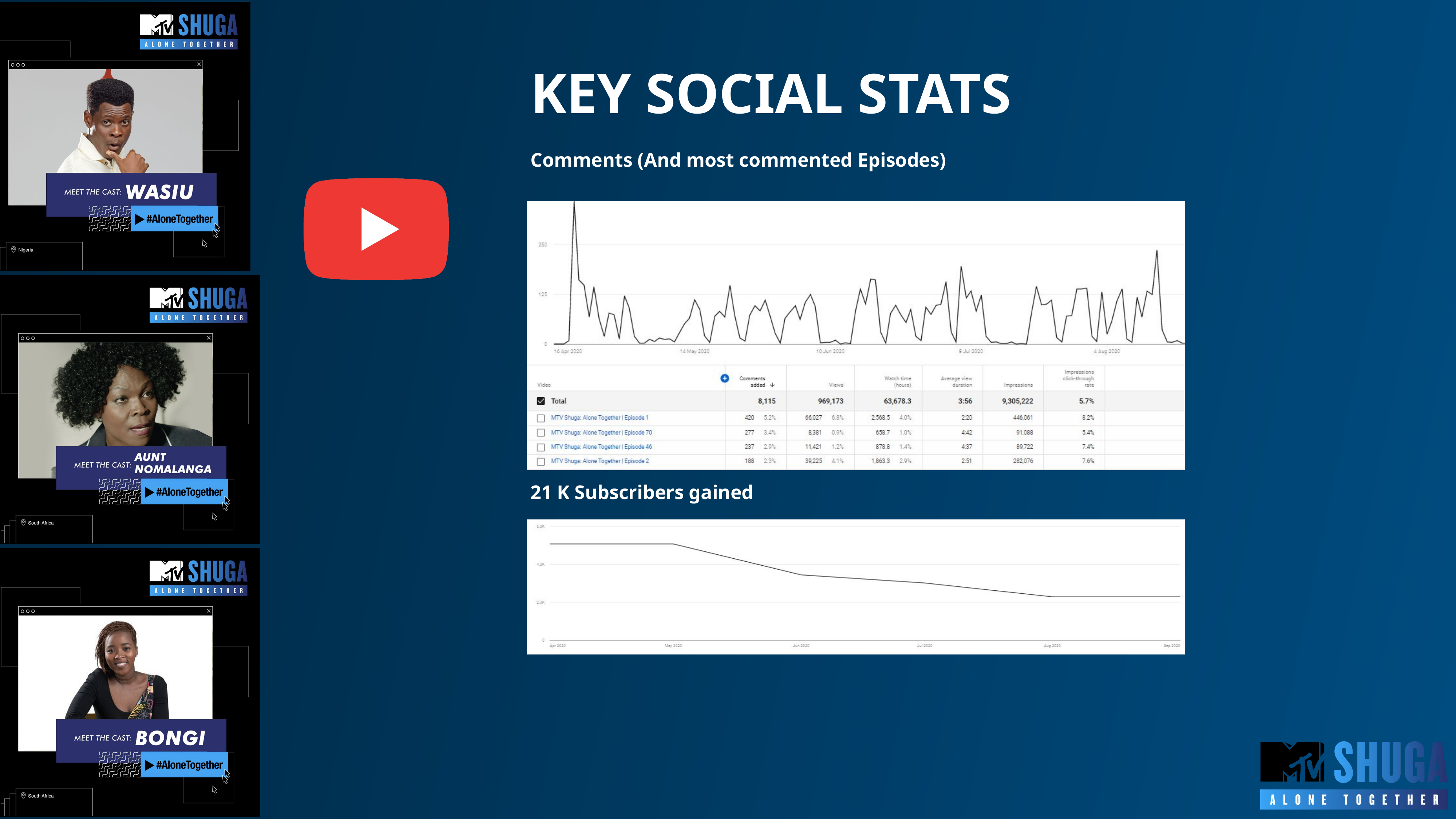

KEY SOCIAL STATS
Comments (And most commented Episodes)
21 K Subscribers gained

## Slide 15
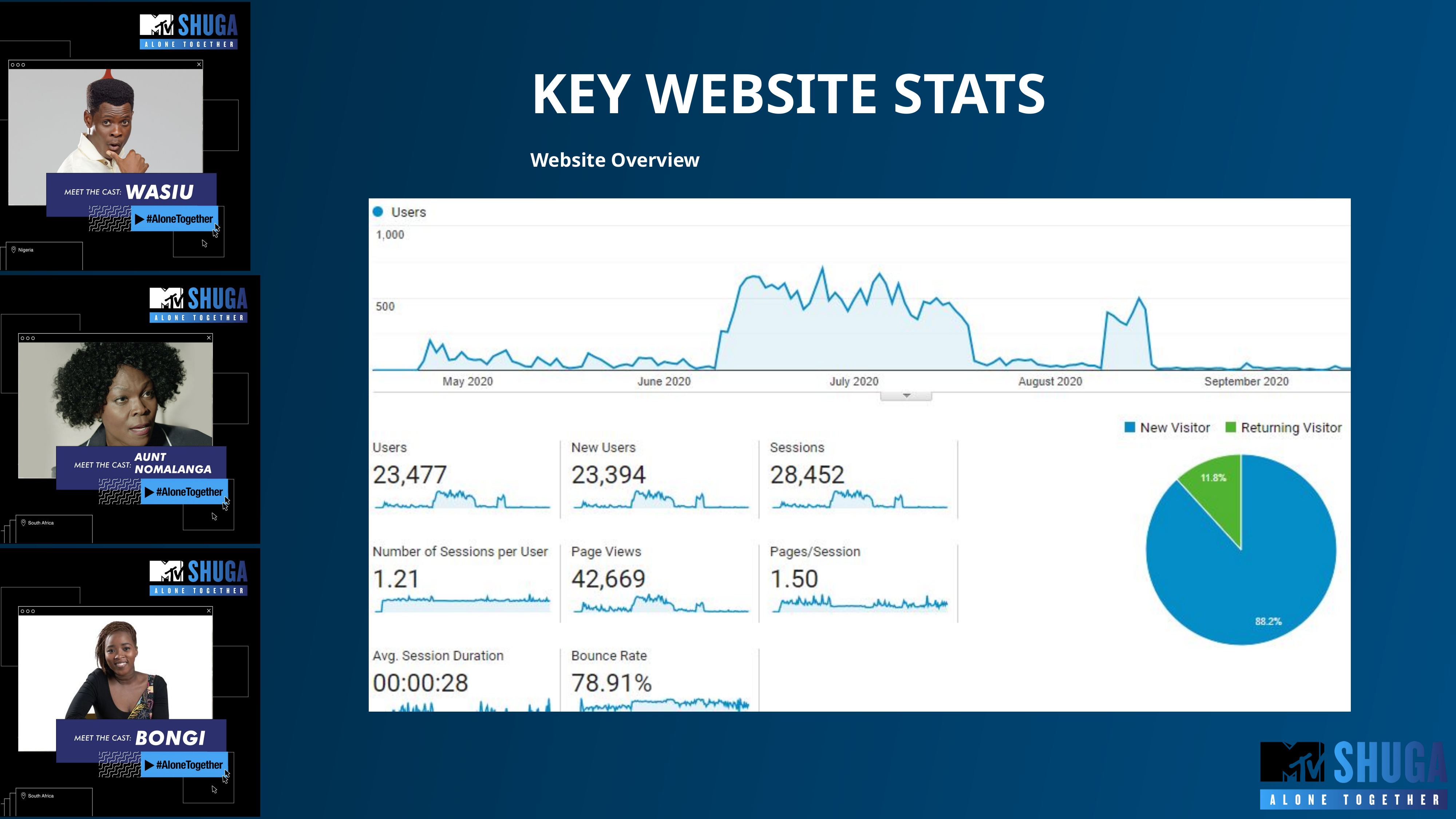

KEY WEBSITE STATS
Website Overview

## Slide 16
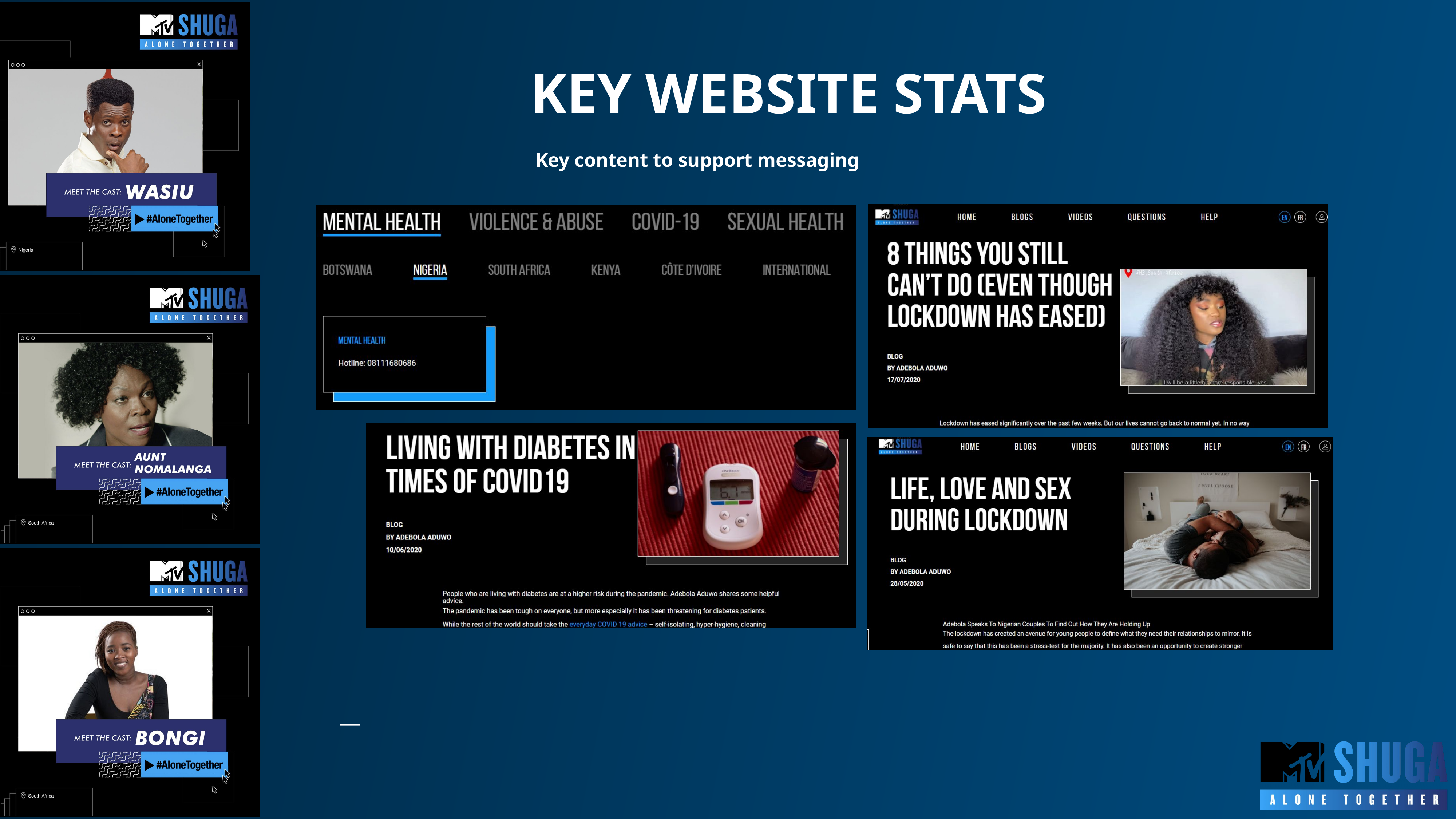

KEY WEBSITE STATS
 Key content to support messaging
__

## Slide 17
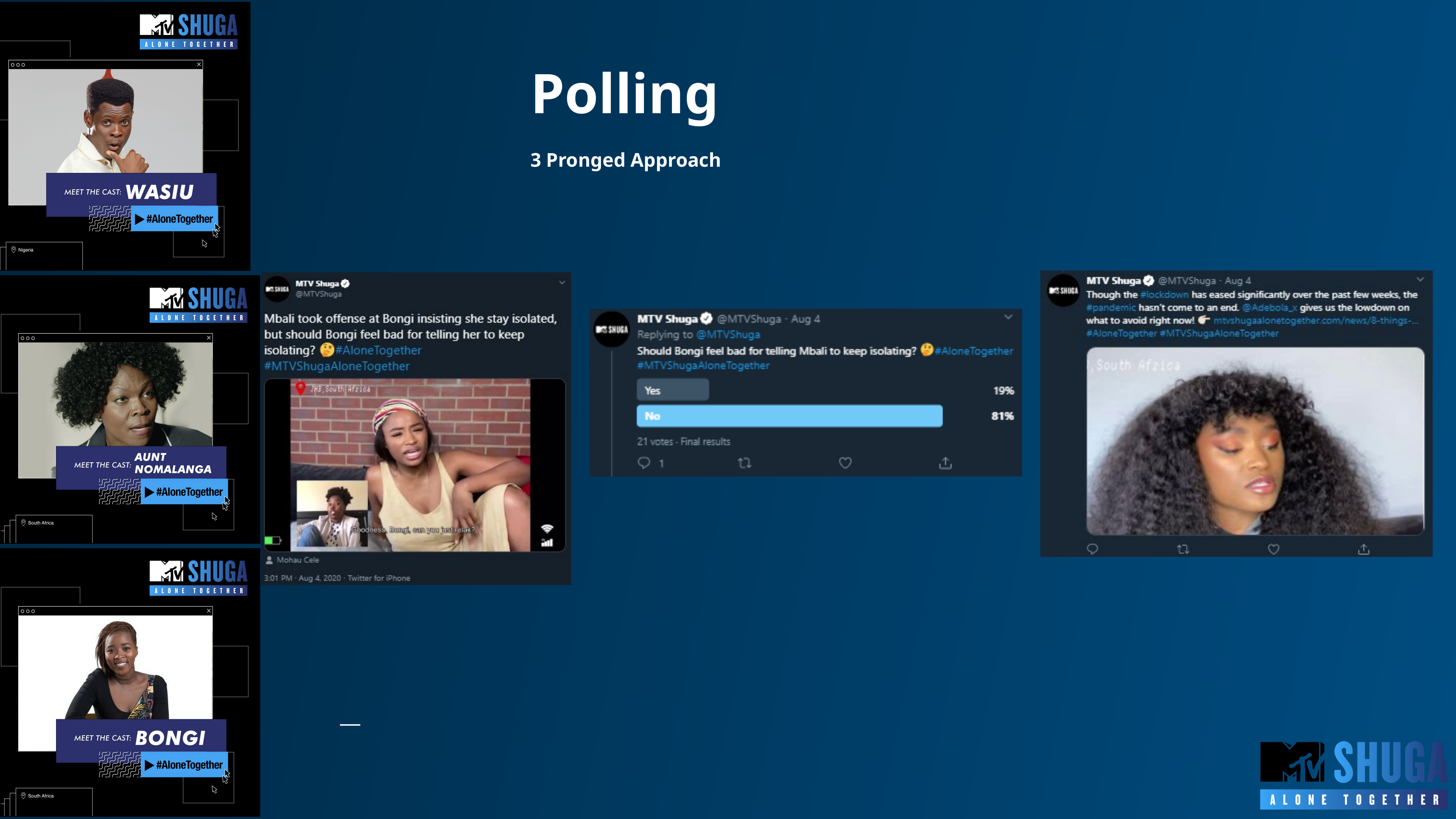

Polling
3 Pronged Approach
__

## Slide 18
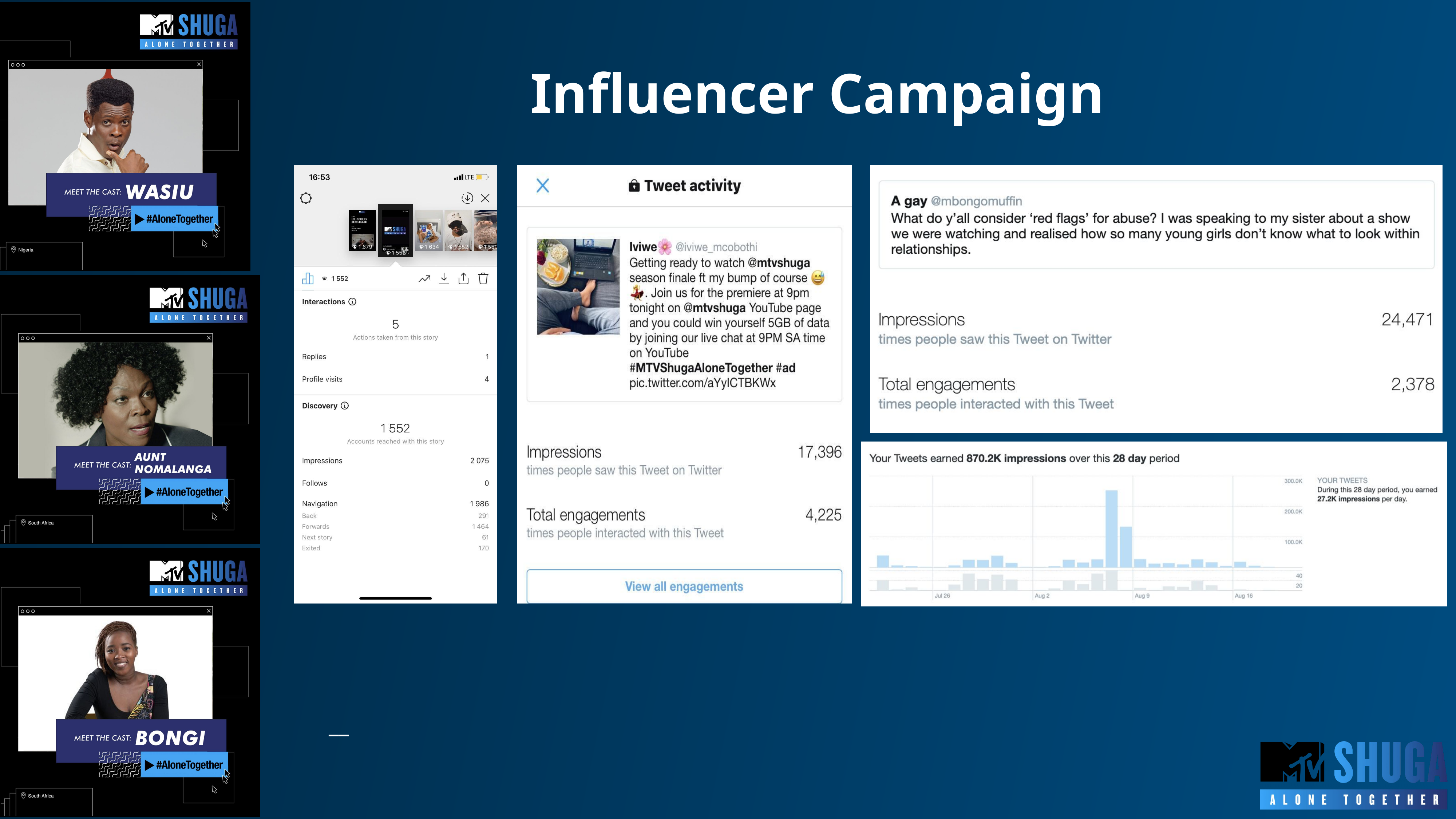

Influencer Campaign
__

## Slide 19
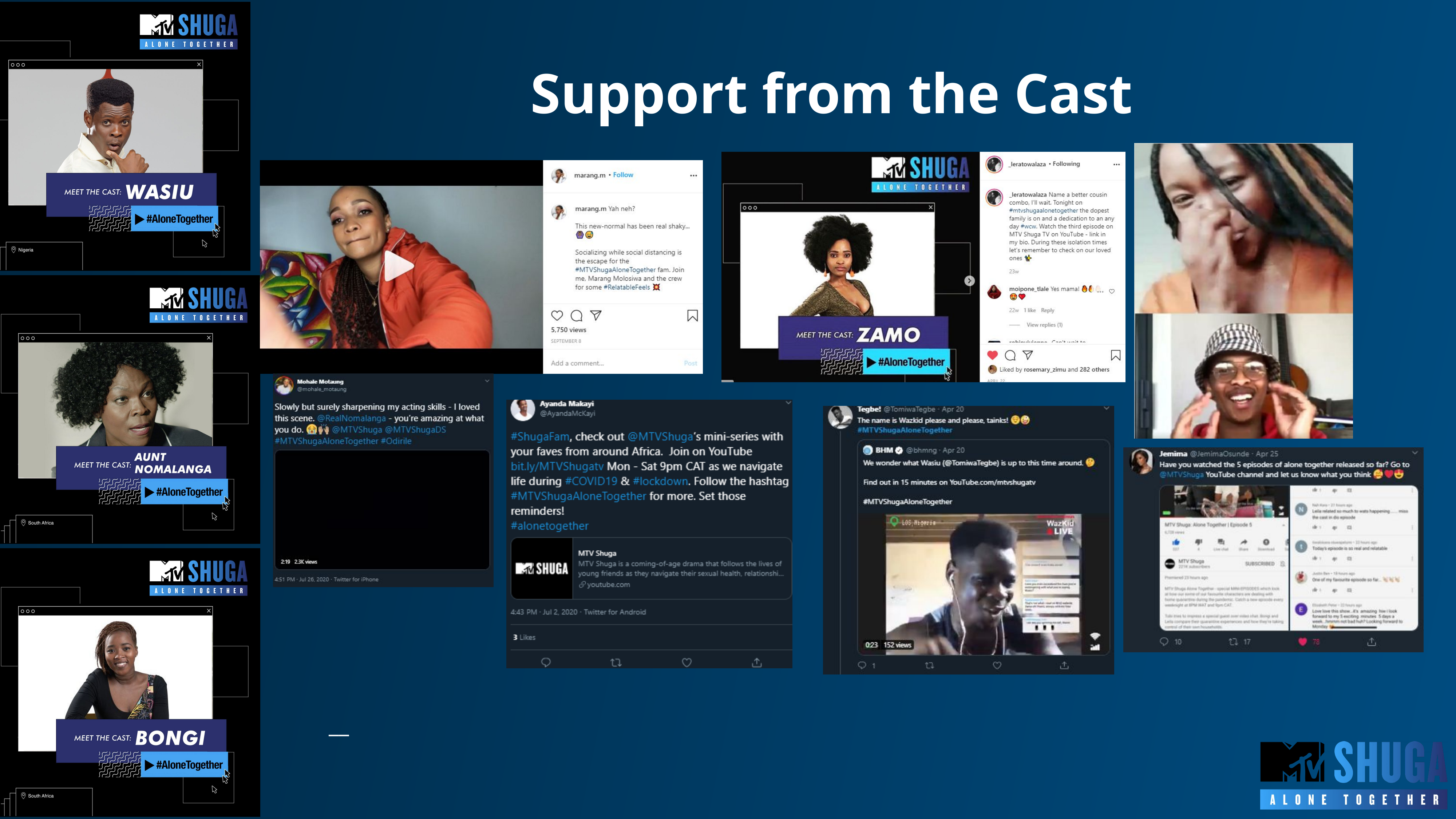

Support from the Cast
__

## Slide 20
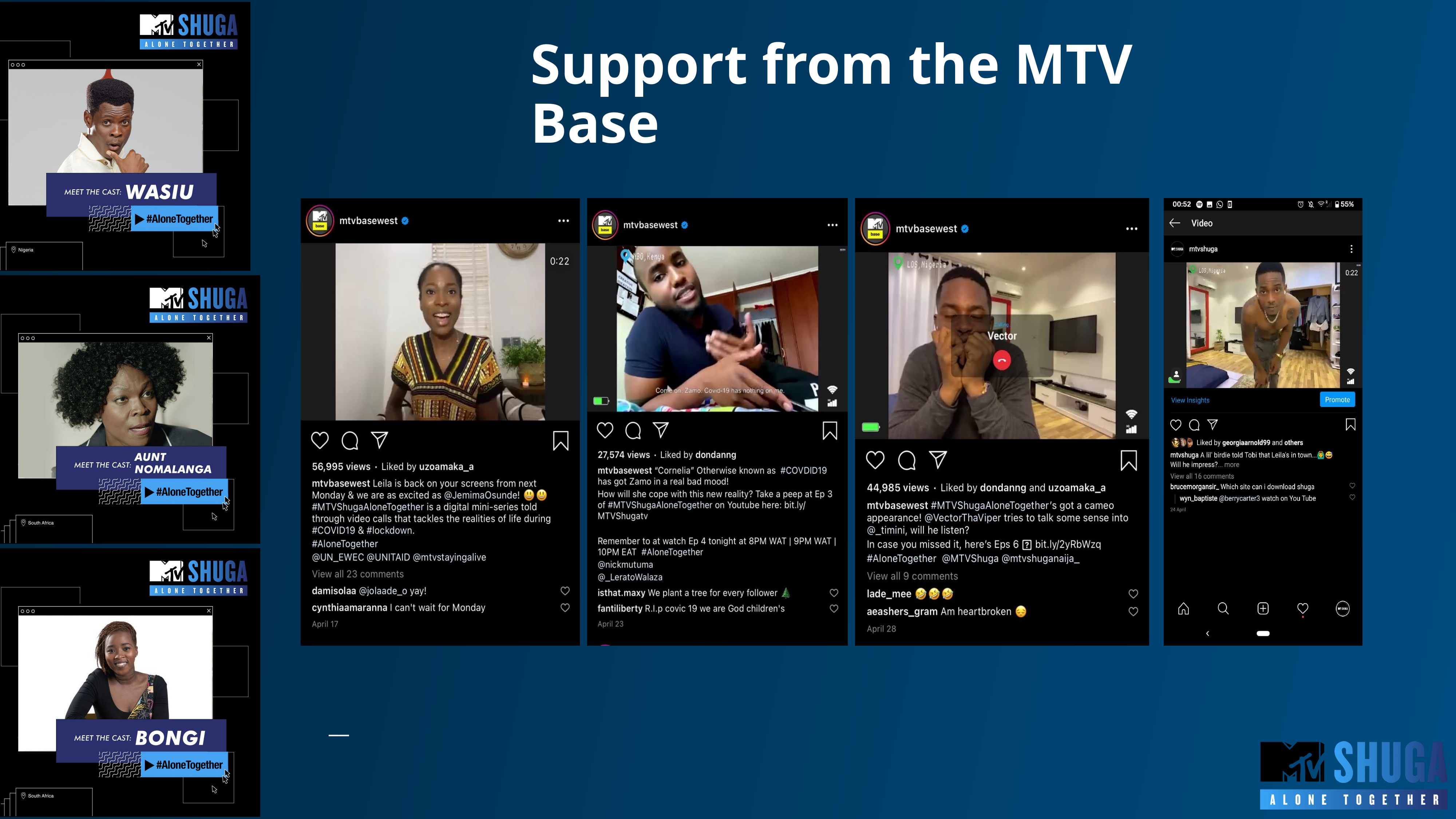

Support from the MTV Base
__

## Slide 21
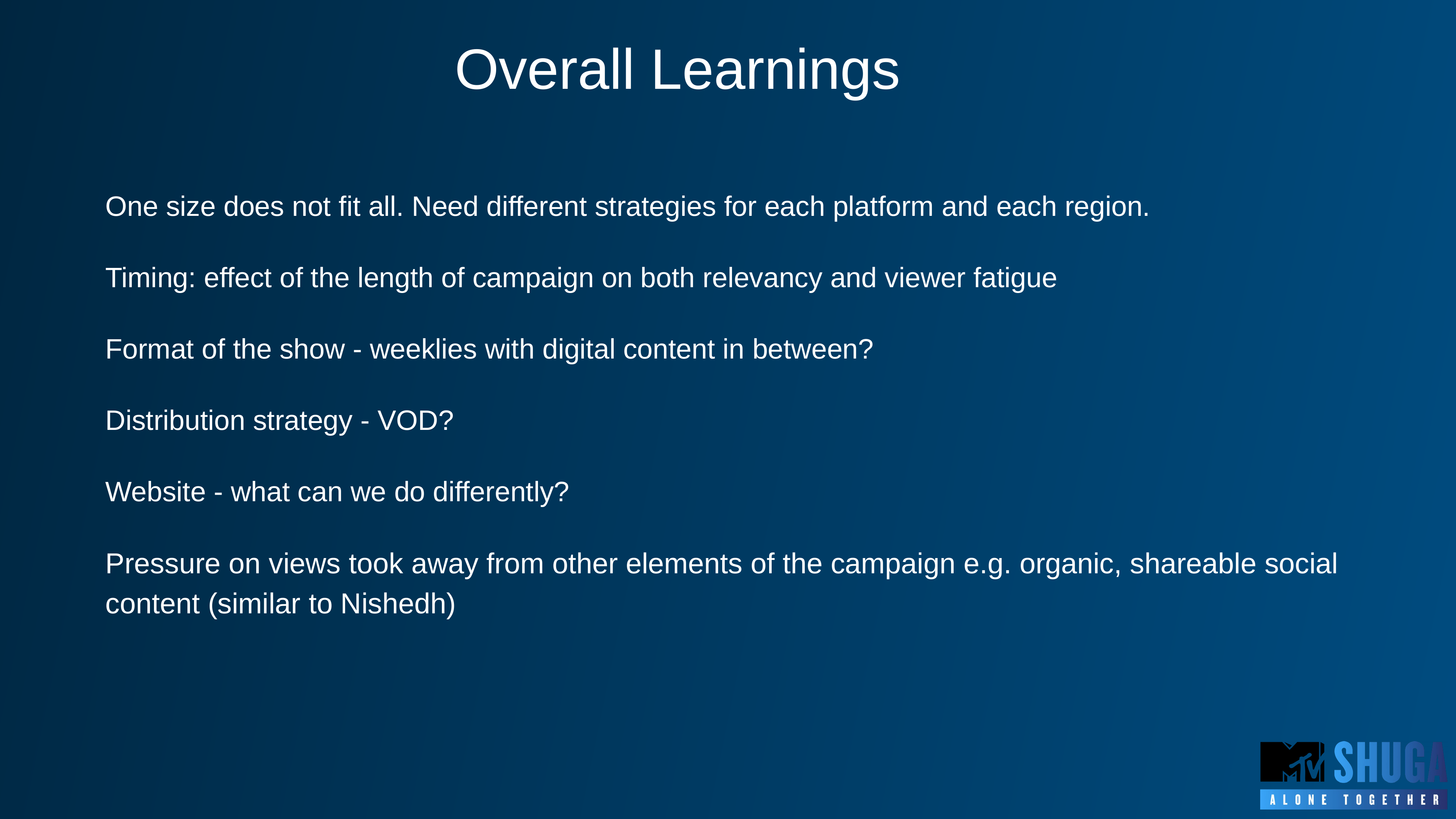

Overall Learnings
One size does not fit all. Need different strategies for each platform and each region.
Timing: effect of the length of campaign on both relevancy and viewer fatigue
Format of the show - weeklies with digital content in between?
Distribution strategy - VOD?
Website - what can we do differently?
Pressure on views took away from other elements of the campaign e.g. organic, shareable social content (similar to Nishedh)

## Slide 22
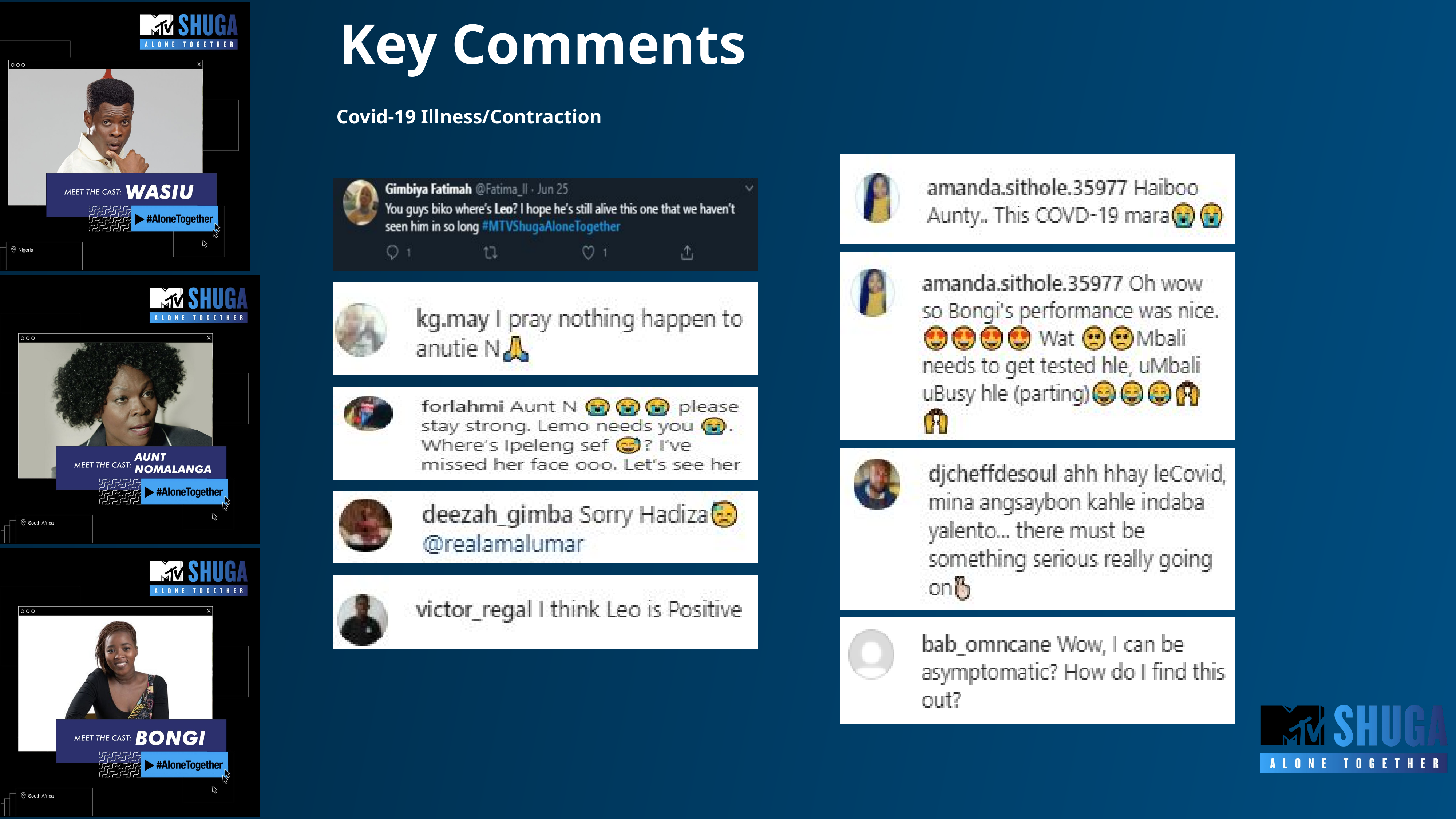

Key Comments
Covid-19 Illness/Contraction

## Slide 23
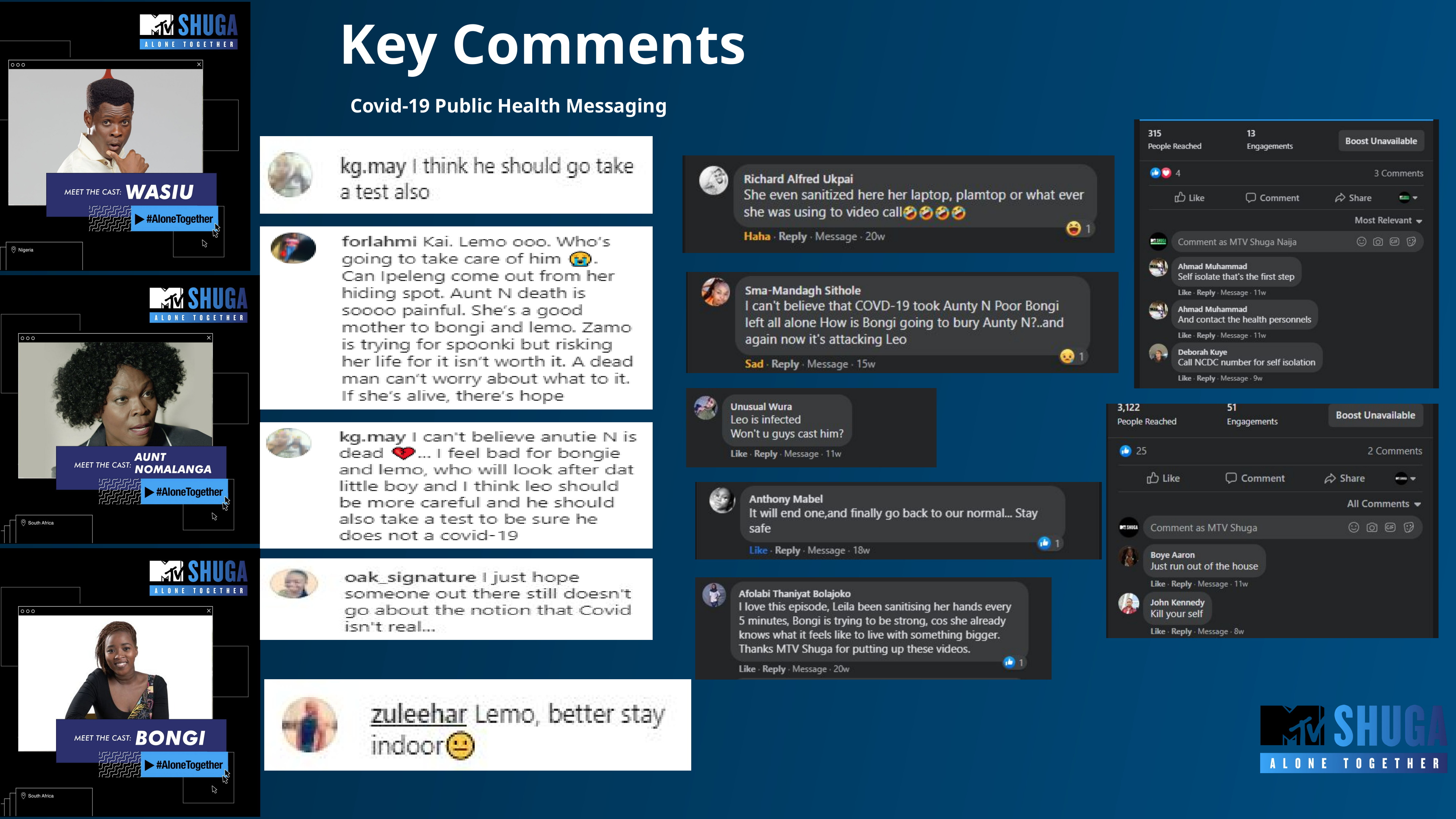

Key Comments
Covid-19 Public Health Messaging

## Slide 24
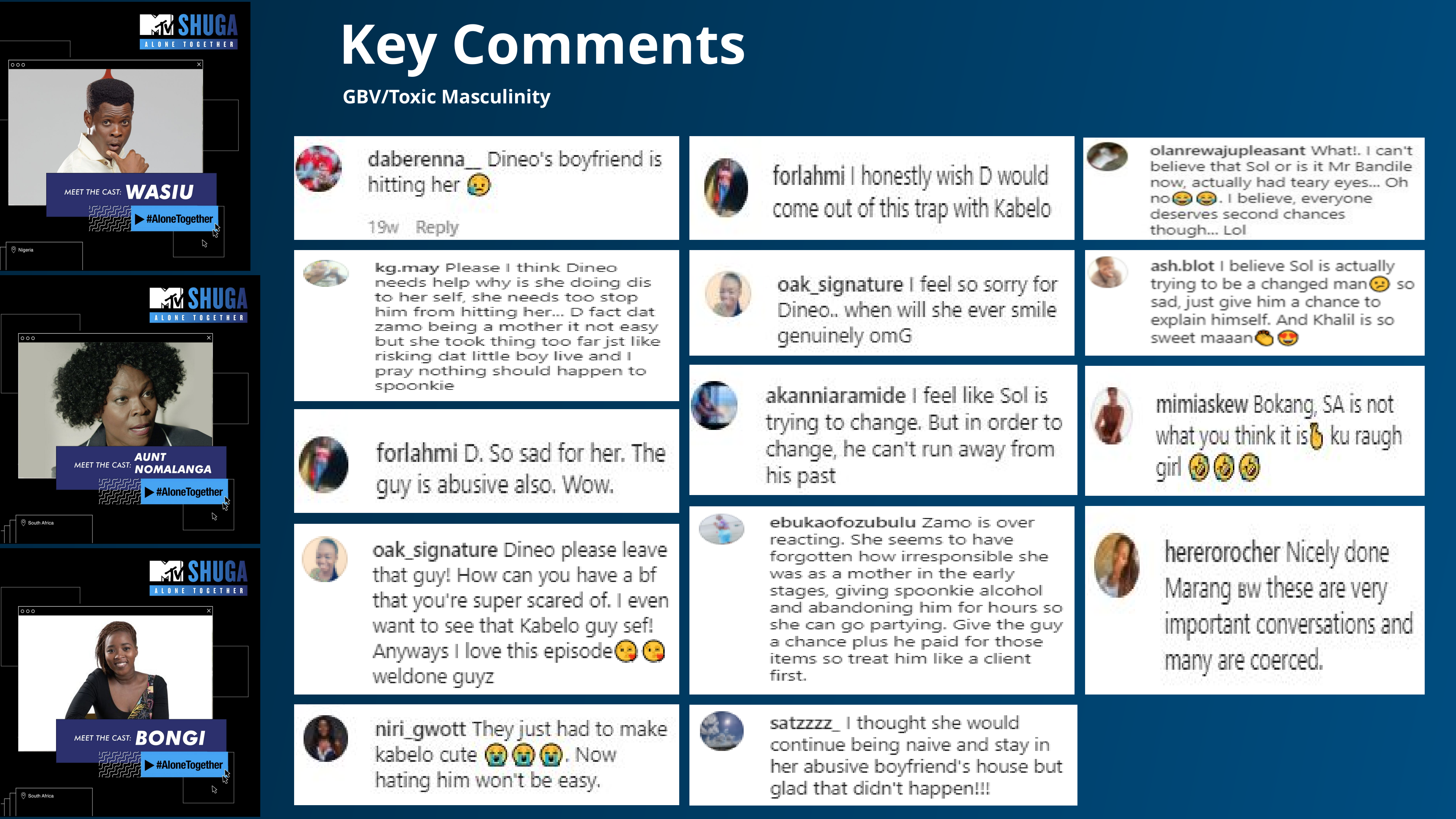

Key Comments
GBV/Toxic Masculinity

## Slide 25
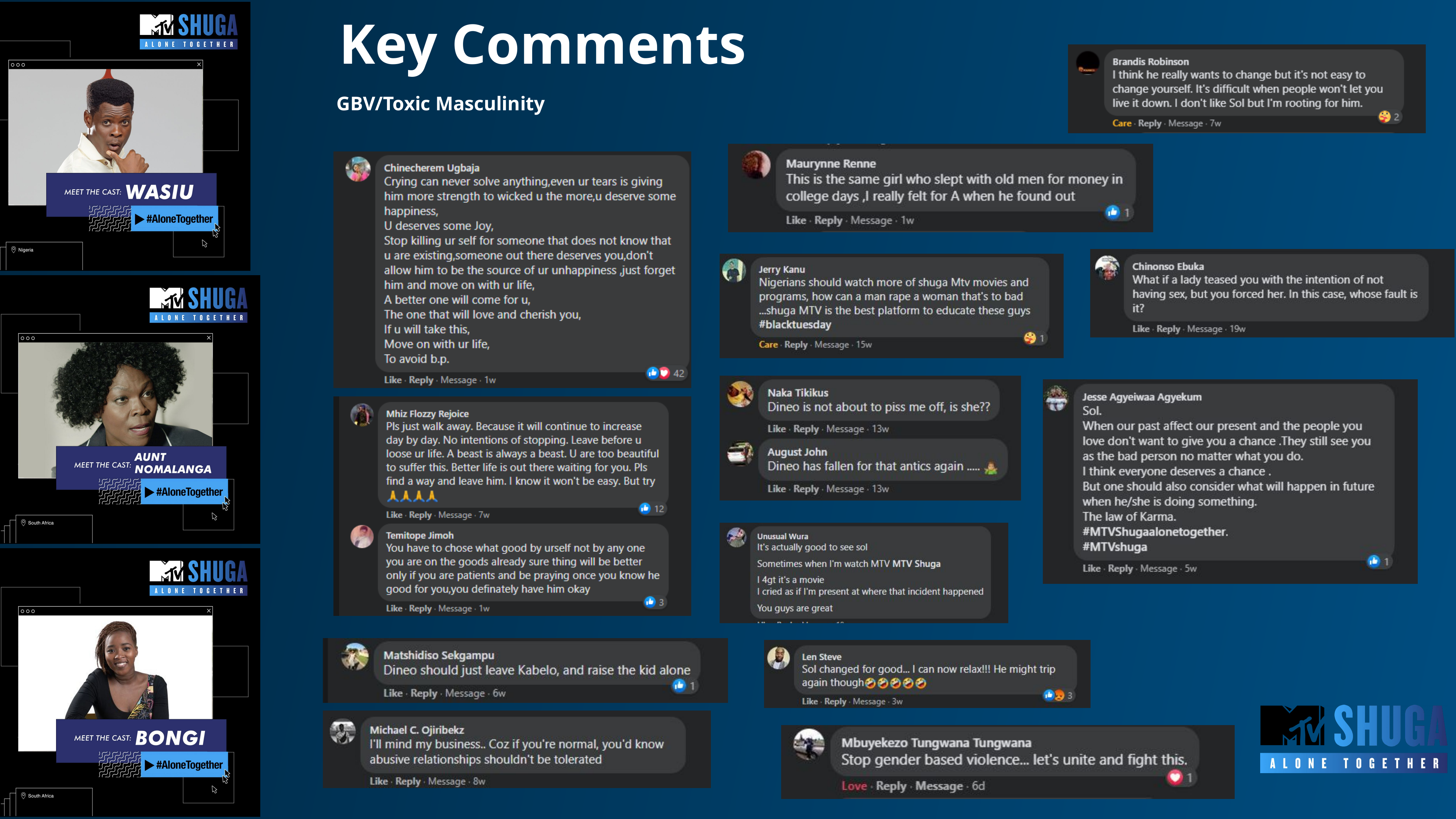

Key Comments
GBV/Toxic Masculinity

## Slide 26
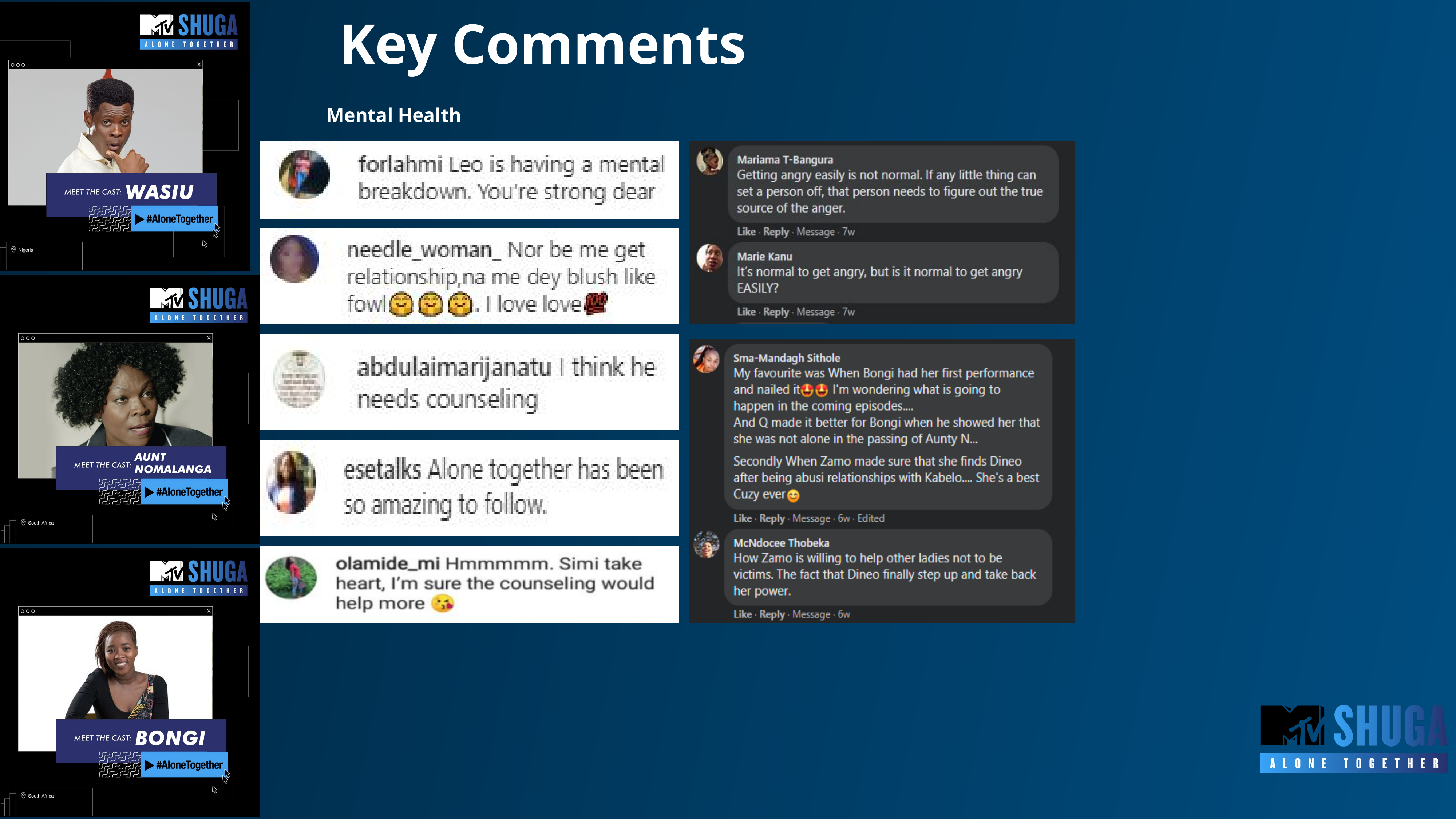

Key Comments
Mental Health

## Slide 27
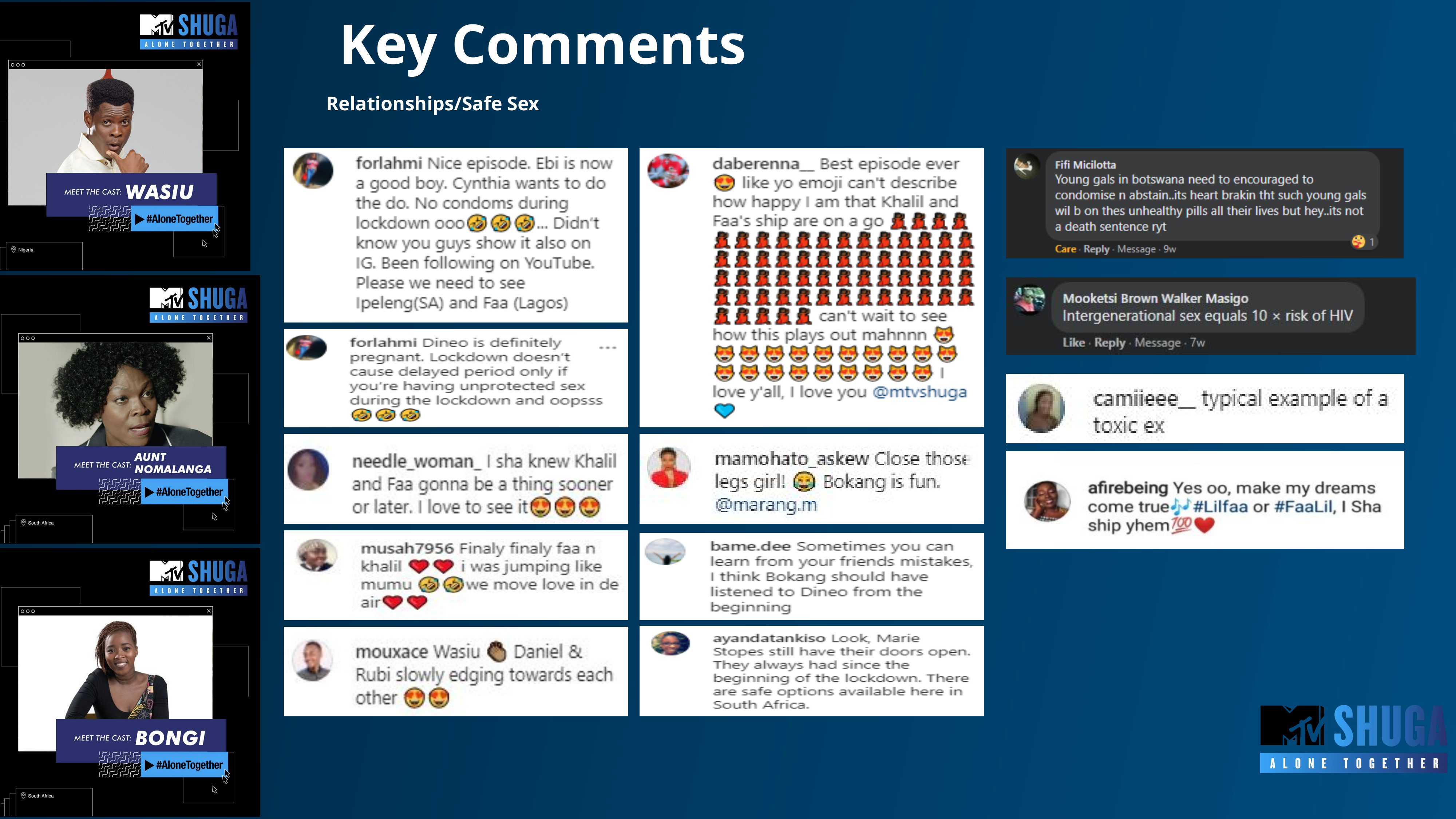

Key Comments
Relationships/Safe Sex

## Slide 28
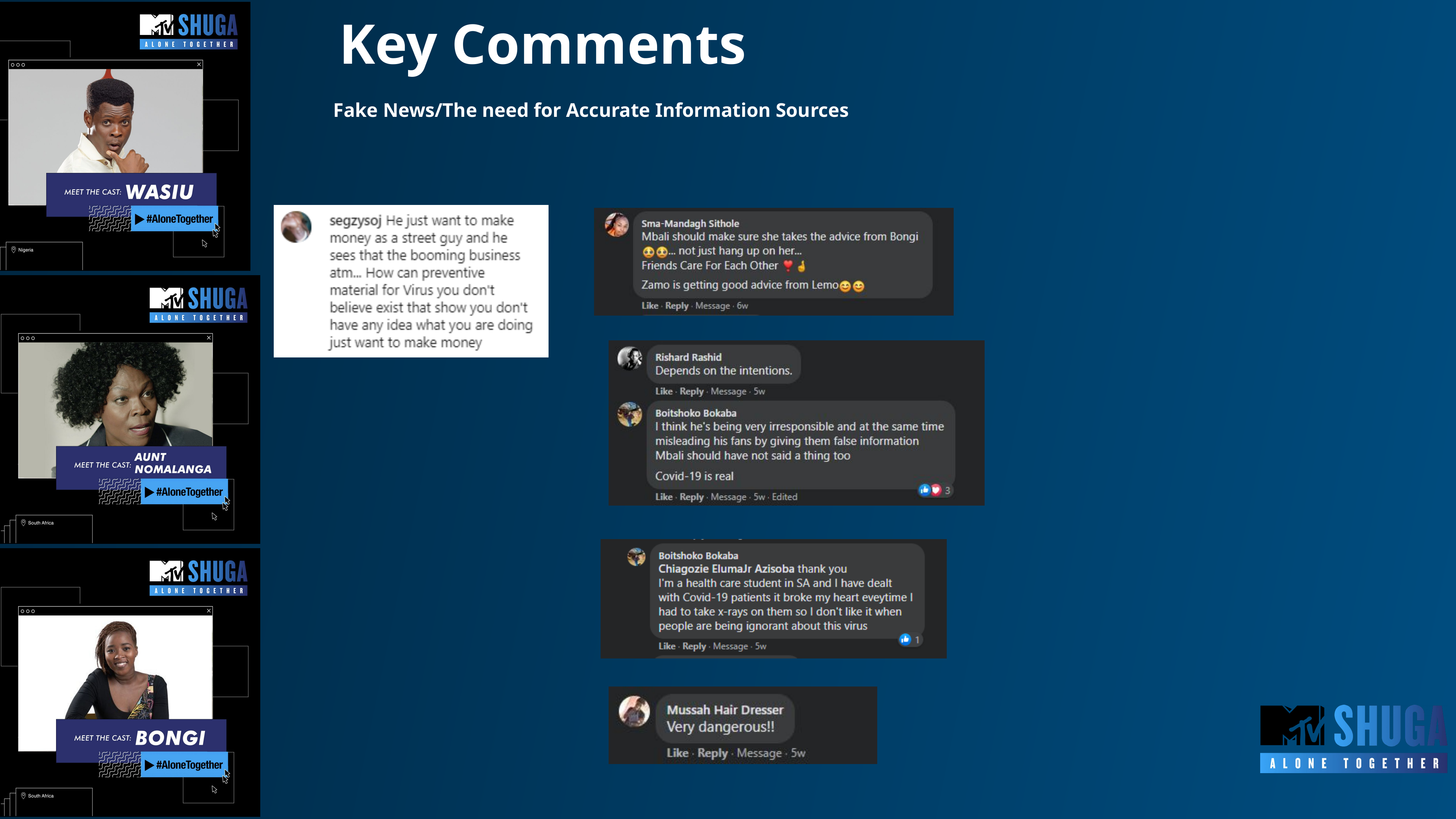

Key Comments
Fake News/The need for Accurate Information Sources

## Slide 29
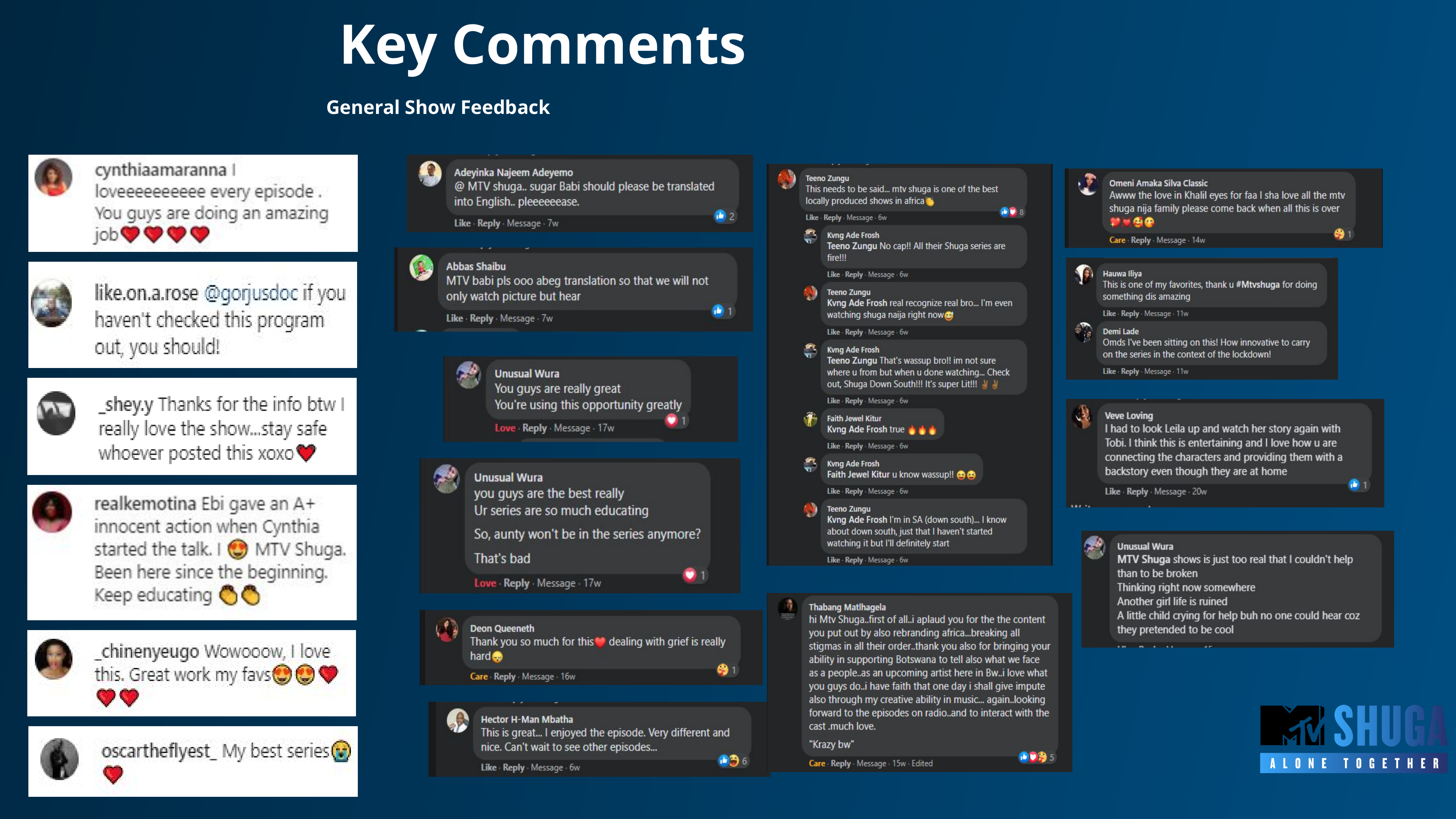

Key Comments
General Show Feedback

## Slide 30
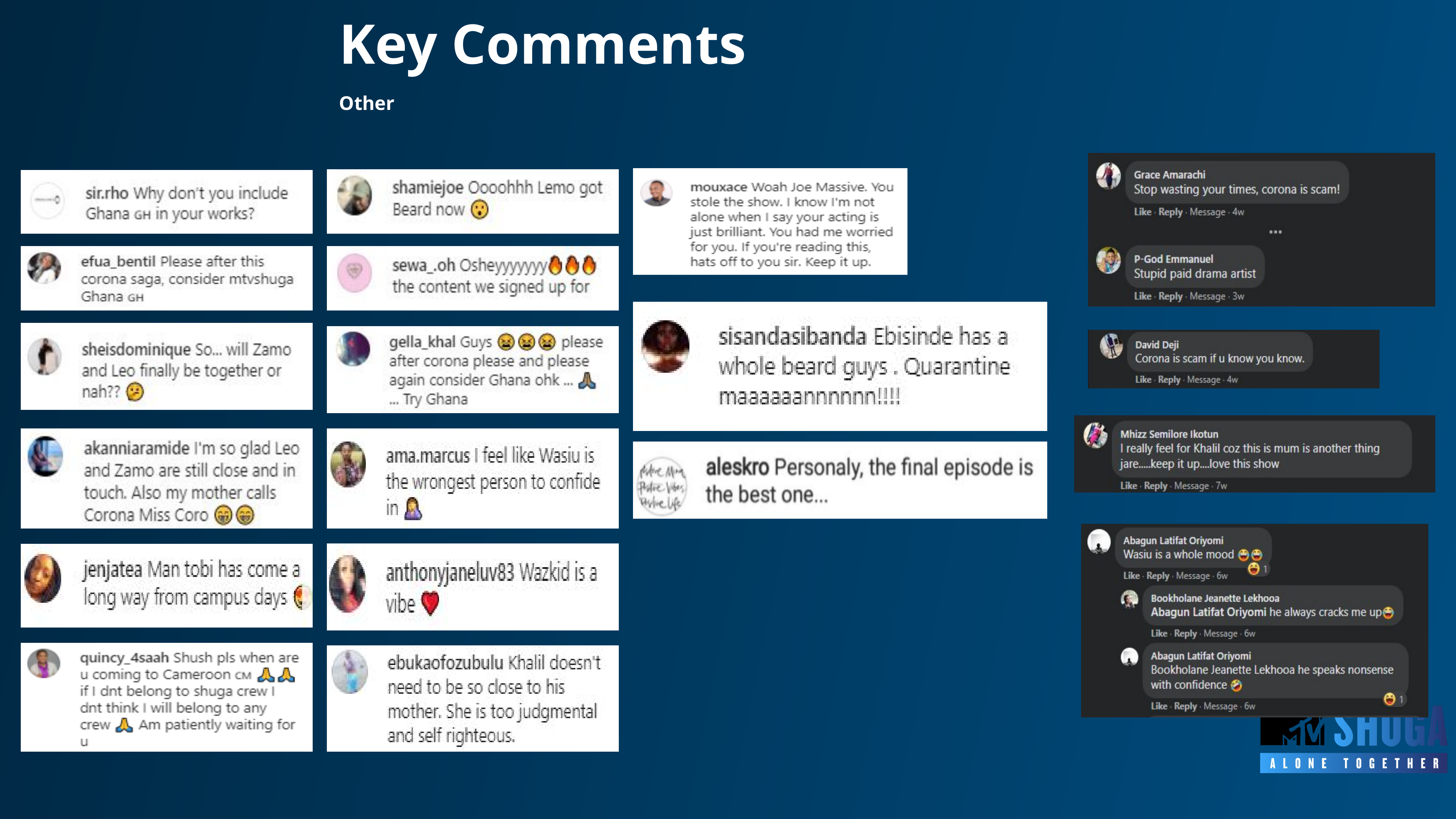

Key Comments
Other
